# 1,4-dihydroxy quininib modulates the secretome of uveal melanoma tumour explants and a marker of oxidative phosphorylation in a metastatic xenograft model

**DOI:** 10.3389/fmed.2022.1036322

**Published:** 2023-01-09

**Authors:** Kayleigh Slater, Rosa Bosch, Kaelin Francis Smith, Chowdhury Arif Jahangir, Sandra Garcia-Mulero, Arman Rahman, Fiona O’Connell, Josep M. Piulats, Valerie O’Neill, Noel Horgan, Sarah E. Coupland, Jacintha O’Sullivan, William M. Gallagher, Alberto Villanueva, Breandán N. Kennedy

**Affiliations:** ^1^UCD School of Biomolecular and Biomedical Science, University College Dublin, Dublin, Ireland; ^2^UCD Conway Institute of Biomolecular and Biomedical Research, University College Dublin, Dublin, Ireland; ^3^Xenopat S.L., Parc Científic de Barcelona, Barcelona, Spain; ^4^Unit of Biomarkers and Susceptibility, Oncology Data Analytics Program (ODAP), Catalan Institute of Oncology (ICO), Oncobell Program, Bellvitge Biomedical Research Institute (IDIBELL) and CIBERESP, L’Hospitalet de Llobregat, Barcelona, Spain; ^5^Department of Clinical Sciences, Faculty of Medicine and Health Sciences, University of Barcelona, Barcelona, Spain; ^6^Department of Surgery, Trinity St. James’s Cancer Institute, Trinity Translational Medicine Institute, St. James’s Hospital, Dublin, Ireland; ^7^Department of Medical Oncology, Catalan Institute of Cancer (ICO), Bellvitge Biomedical Research Institute (IDIBELL)-OncoBell, Barcelona, Spain; ^8^Royal Victoria Eye and Ear Hospital, Dublin, Ireland; ^9^Liverpool Ocular Oncology Research Group, Department of Molecular and Clinical Cancer Medicine, Institute of Systems, Molecular and Integrative Biology, University of Liverpool, Liverpool, United Kingdom; ^10^Chemoresistance and Predictive Factors Group, Program Against Cancer Therapeutic Resistance (ProCURE), Catalan Institute of Oncology (ICO), Oncobell Program, Bellvitge Biomedical Research Institute (IDIBELL), L’Hospitalet de Llobregat, Barcelona, Spain

**Keywords:** cysteinyl leukotriene, uveal melanoma (UM), xenograft model, tumour metabolism, inflammation, immunohistochemistry, tumour microenvironment, ATP5B ATP synthase

## Abstract

Uveal melanoma (UM) is an intraocular cancer with propensity for liver metastases. The median overall survival (OS) for metastatic UM (MUM) is 1.07 years, with a reported range of 0.84–1.34. In primary UM, high cysteinyl leukotriene receptor 1 (CysLT_1_) expression associates with poor outcomes. CysLT_1_ antagonists, quininib and 1,4-dihydroxy quininib, alter cancer hallmarks of primary and metastatic UM cell lines *in vitro*. Here, the clinical relevance of CysLT receptors and therapeutic potential of quininib analogs is elaborated in UM using preclinical *in vivo* orthotopic xenograft models and *ex vivo* patient samples. Immunohistochemical staining of an independent cohort (*n* = 64) of primary UM patients confirmed high CysLT_1_ expression significantly associates with death from metastatic disease (*p* = 0.02; HR 2.28; 95% CI 1.08–4.78), solidifying the disease relevance of CysLT_1_ in UM. In primary UM samples (*n* = 11) cultured as *ex vivo* explants, 1,4-dihydroxy quininib significantly alters the secretion of IL-13, IL-2, and TNF-α. In an orthotopic, cell line-derived xenograft model of MUM, 1,4-dihydroxy quininib administered intraperitoneally at 25 mg/kg significantly decreases ATP5B expression (*p* = 0.03), a marker of oxidative phosphorylation. In UM, high *ATP5F1B* is a poor prognostic indicator, whereas low *ATP5F1B*, in combination with disomy 3, correlates with an absence of metastatic disease in the TCGA-UM dataset. These preclinical data highlight the diagnostic potential of CysLT_1_ and *ATP5F1B* in UM, and the therapeutic potential of 1,4-dihydroxy quininib with *ATP5F1B* as a companion diagnostic to treat MUM.

## Introduction

Uveal melanoma (UM) is a rare, intraocular cancer that arises from melanocytes within the uveal tract, consisting of the choroid, ciliary body, and iris. UM impacts patients by threatening visual impairment, ocular discomfort, and in up to half of all cases, death from metastatic disease (1, 2). Despite major advances and improvements in control of local eye disease, it remains unclear whether ocular treatment influences patient outcomes (3), and there are very limited therapeutic options available once metastases are detected. At present, many patients will undergo severe, life-altering enucleation surgery or radiotherapy without any clear indication that it will reduce their risk of metastatic disease or prolong their lives (3). Through hematogenous spread, UM metastasizes in up to 50% of patients diagnosed with primary UM (4). The liver is the predominant site for secondary disease, and once metastases are detected, the outlook for patients is extremely somber. The median overall survival is approximately 13.4 months, with as few as 8% of patients surviving beyond 2 years (5–7). In 2022, tebentafusp (kimmtrak), a bispecific fusion protein that redirects CD3 + T cells to gp100-expressing melanoma cells (8), received FDA and EMA approval as the first drug to improve overall survival in metastatic UM. In a phase III trial of 378 patients, tebentafusp achieved a 1-year overall survival (OS) rate of 73.2 vs. 58.5% in the investigator’s choice arm (8). This translates to a gain in median overall survival of 5.7 months in the tebentafusp treatment group (8). Tebentafusp specifically targets cells presenting the HLA-A*02:01 subtype and is not effective in HLA-A*02:01 negative patients (2). One limitation therefore, is that only 50% of Caucasians, in whom UM is most prevalent (9), are HLA-A*02:01 positive and eligible for tebentafusp treatment (10). Despite this advancement in MUM treatment, there remains a lack of therapies for HLA-A*02:01 negative patients, or therapies that can halt or prevent the progression of UM metastases.

There is increasing interest in determining the role of cysteinyl leukotrienes (CysLTs), and their associated G-protein coupled receptors (CysLT_1_ and CysLT_2_), in promoting tumorigenesis in various cancers (11, 12). CysLTs may contribute to a shift toward a tumour promoting microenvironment (12, 13), and upregulation of CysLT receptors and altered CysLT production is documented in several cancers, including colorectal cancer, prostate cancer, renal cell carcinoma, transitional cell carcinoma and testicular cancer (14–17). Using the TCGA-UM cohort of 80 patients with primary UM, we previously identified high expression of *CYSLTR1* or *CYSLTR2* as significantly associated with reduced disease-specific and overall survival (18). In a cohort of 52 UK patients presenting with primary UM, high immunohistochemical expression of CysLT_1_ was significantly associated with reduced survival from metastatic disease and reduced overall survival, as assessed by both manual and digital pathology analysis (18). CysLT_1_ expression in primary UM is also significantly associated with ciliary body involvement (18), an established indicator of poor prognosis in UM (19, 20). Thus, our previous data suggests high expression of CysLT_1_ in primary UM is significantly associated with a poor prognosis.

CysLT receptor antagonism as an anti-cancer strategy is not a new concept, indeed CysLT_1_ antagonists are reported to exert anti-cancer properties across several cancer types (16, 21–24). Separately, a large, population-based study in newly diagnosed asthmatic patients found that CysLT_1_ antagonists decreased the risk of 14 different cancers by 60–78% in a dose-dependent manner (25). Owing to the established link between chronic inflammation and colorectal cancer (CRC), a large body of work has focused on the link between CysLTs and CRC (26, 27). For example, 1,4-dihydroxy quininib alters the secretion of inflammatory and angiogenic factors from *ex vivo* CRC tumour explants and reduces the growth of tumours in a cell line-derived xenograft model of CRC *in vivo* (28, 29). In UM, we were the first to demonstrate the anti-cancer potential of the CysLT_1_ antagonists *in vitro*. Quininib and 1,4-dihydroxy quininib significantly alter viability, long-term proliferation, secretion of inflammatory and angiogenic factors, and oxidative phosphorylation in primary and metastatic UM cell lines (18). Similarly, CysLT_1_ antagonists significantly inhibit tumour growth in *in vivo* orthotopic zebrafish xenograft models of UM (18). In contrast, the CysLT_2_ specific antagonist, HAMI 3379, had negligible effects on UM cell lines in all *in vitro* assays examined (18). Our previously published *in vitro* data shows that montelukast is less effective than quininib or 1,4-dihydroxy quininib at altering the tumorigenic properties of UM cell lines (18). Quininib drugs outperform montelukast in UM cell viability assays and long-term proliferation assays (18). Similarly, montelukast had no significant effect on the cancer secretome of inflammatory or angiogenic mediators from UM cells. Based on these findings, we chose to progress the quininib drugs, and not montelukast for translational studies.

Here, we further interrogate the anti-cancer potential of the quininib CysLT_1_ antagonists in more clinically relevant models of primary and metastatic UM. We verify, using established cut-off values, that high expression of CysLT_1_ is significantly associated with reduced survival from metastatic disease and reduced overall survival in a second, independent validation cohort of patients with primary UM. In primary UM patient samples grown as explant cultures, secretion of inflammatory and angiogenic factors from vehicle and 1,4-dihydroxy quininib treated tumours significantly correlated with clinical features of UM. Similarly, the secretion of IL-2, IL-13, and TNF-α is significantly higher in tumour-conditioned media (TCM) derived from 1,4-dihydroxy quininib treated primary UM tumours vs. vehicle. CysLT_1_ antagonists alter MAPK signaling in Mel285 primary UM cells, as reported in other cancer and non-cancer cell types (21, 30, 31), but not in metastatic OMM2.5 UM cells bearing a G_*aq*_ Q209P mutation that constitutively activates ERK (32). In a cell line-derived orthotopic xenograft model of MUM, treatment with 1,4-dihydroxy quininib did not significantly reduce tumour weight vs. vehicle following 3 weeks of treatment. However, expression of ATP5B, a protein marker of oxidative phosphorylation, was significantly reduced in 1,4-dihydroxy quininib treated mice. Furthermore, high expression of *ATP5F1B* in primary UM is significantly associated with reduced progression-free survival and reduced overall survival (OS). Interestingly, we found a significant difference in disease free survival in disomy 3 patients combined with high vs. low *ATP5F1B* expression. This suggests that patients with disomy 3 and low *ATP5F1B* expression have a reduced risk of metastatic disease vs. patients with disomy 3 and high *ATP5F1B.* This preclinical data solidifies the role of CysLT_1_ expression as a poor prognostic indicator and offers prognostic potential for *ATP5F1B* in combination with disomy 3 in primary UM tumours.

## Materials and methods

### Ethics

This study conformed to the principles of the Declaration of Helsinki and Good Clinical Practice guidelines. Ethical approval for samples included in the tissue microarray was obtained from Hospital de Bellvitge clinical research ethics committee. All patients provided written, informed consent prior to involvement. Ethical approval to obtain primary uveal melanoma tumour tissue post enucleation was granted by the Royal Victoria Eye and Ear Hospital on 27 November 2018. All patients involved in this study provided written, informed consent. Exemption from full ethical review was granted by the UCD research ethics committee on 19 February 2019 under reference number LS-E-19-23-Slater-Kennedy. All experiments involving the use of rodents were approved by the Ethical Committee of Animal Experimentation of the Parc Científic de Barcelona (PCB) under the procedure number 9928-P1 approved by the Generalitat de Catalunya.

### Immunohistochemistry of primary uveal melanoma TMA

**FIGURE 1:**
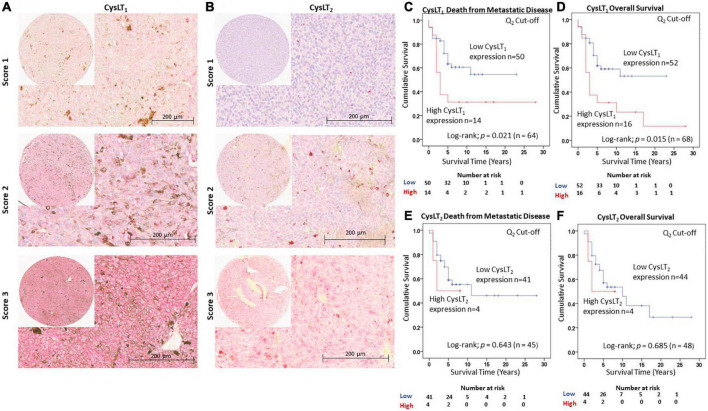
Validation of the prognostic value of CysLT_1_ and CysLT_2_ protein expression in an independent cohort of primary UM patients. **(A)** Representative cores from the Spanish UM patient tissue microarray designated with a score of 1, 2, or 3 for CysLT_1_ staining intensity. **(B)** Representative cores from the same TMA designated with a score of 1, 2, or 3 for CysLT_2_ staining intensity. Expression of both CysLT_1_ and CysLT_2_ was predominantly cytoplasmic in primary UM tumours, with a mixture of membranous and cytoplasmic staining in cores designated a score of 2 and 3. **(C)** High expression of CysLT_1_ (red) is significantly associated with reduced survival from metastatic disease in patients presenting with primary UM (*n* = 64; Log Rank; *p* = 0.021; HR 2.28; 95% CI 1.08–4.78). **(D)** High expression of CysLT_1_ (red) is significantly associated with reduced overall survival in patients presenting with primary UM (*n* = 68; Log Rank; *p* = 0.015; HR 2.27; 95% CI 1.12–4.58). **(E)** Kaplan-Meier survival curve stratified based on high (red) or low (blue) CysLT_2_ expression and death by metastatic melanoma (*n* = 45; Log Rank; *p* = 0.643; HR 1.39; 95% CI 0.32–6.01). **(F)** Kaplan-Meier survival curve stratified based by high (red) or low (blue) CysLT_2_ expression and death by any cause (*n* = 48; Log Rank; *p* = 0.685; HR 1.34; 95% CI 0.31–5.75). The median was used as the cut-off point for high vs. low expression for all Kaplan-Meier survival curves. Number of events indicates the number of deaths due to metastatic melanoma **(C,E)**. Number of events indicates the number of deaths due to any cause **(D,F)**.

Immunohistochemistry (IHC) for CysLT_1_ (Abcam—ab151484, 1:200) and CysLT_2_ (Cayman Chemical–CAY120560, 1:500) was performed on 4-μm formalin-fixed paraffin-embedded (FFPE) sections arranged on a TMA using commercial equipment (Leica Bond RXm System; Leica Microsystems Ltd., Milton Keynes, United Kingdom) and a detection kit (Bond Polymer Refine Red Detection Kit; Leica Biosystems, Inc., Buffalo Grove, IL, USA) as previously described (33). Slides were counterstained with hematoxylin and mounted using DPX mountant (Sigma-Aldrich, St. Louis, MO, USA). Colorectal cancer tissue served as the positive control; negative control was omission of the primary antibody (Supplementary Figures 1A–C). Slides were scanned using a slide scanner (Aperio CS2; Leica Biosystems, Inc., Buffalo Grove, IL, USA) and analyzed with imaging software (Aperio Image Scope version 11.2; Leica Biosystems, Inc., Buffalo Grove, IL, USA). Each core was scored based on intensity (0–absent, 1–mild, 2–moderate, or 3–intense) (Figures 1A,B) and percentage of tumour cells stained (0 – absent, 1 – 1–24%, 2 – 25–49%, 3 – 50–74%, or 4 – >75%). The final score was calculated using the following equation: (scoring intensity × % of cells stained)/n number of samples (33). The IHC-stained slides were scored by two independent investigators (SEC, KS). Death from metastatic disease is defined as death from metastatic uveal melanoma. Overall survival is defined as death by any cause.

### Explant culture of primary uveal melanoma samples

Human primary UM samples were obtained post enucleation from the Royal Victoria Eye and Ear Hospital, Dublin. Following removal of the eye during surgery, a portion of the tumour was processed, and paraffin embedded for histopathological analysis, another portion was taken for explant culture experiments. Immediately following dissection, the tissue was placed into complete culture medium [RPMI 1640 Medium (Gibco) supplemented with 10% FBS and 2% Penicillin/Streptomycin] at room temperature for transport to University College Dublin. Upon arrival, the tissue was washed three times in sterile PBS wash buffer (PBS and 2% Penicillin/Streptomycin). Using a sterile scalpel and forceps, the tumour specimen was cut into four individual pieces. Samples were incubated in 20 μM quininib, 20 μM 1,4–dihydroxy quininib, 20 μM dacarbazine, or DMSO, made up to 1 ml in complete culture medium in a 12-well plate. Explants were incubated for 72 h at 37°C/5% CO_2_. Plates were wrapped in parafilm to prevent evaporation of medium during the incubation period. After 72 h, the tumour conditioned media was removed. An 800 μl aliquot was stored at −80°C for ELISA analysis and a 200 μl aliquot was stored at 4°C for LDH analysis. The residual explant tissue was immediately snap-frozen in liquid nitrogen and stored at –80°C.

### Drug preparation for use in cell and explant culture

Quininib (Q1), 1,4-dihydroxy quininib (Q7) (30, 34), montelukast (Sigma #SML0101), HAMI 3379 (Cayman Chemical #10580), and dacarbazine (Sigma #D2390) were dissolved in 100% DMSO and stored as (10–50 mM) stock solutions. Working solutions (100 μM) were prepared fresh prior to each experiment in complete cell culture medium as described above. Drugs were made to final test concentrations by adding the required volume of the working solution to cells in complete media. 0.5% DMSO was used as vehicle control.

### Explant total protein determination

Total protein was extracted from each piece of tumour explant tissue. Each individual explant was placed in a tube with 200 μl of ice-cold T-PER lysis reagent (Thermo Fisher Scientific, Rockford, IL, USA) supplemented with 10 μl/ml protease inhibitor and a 3 mm stainless steel bead. Tubes were placed in a TissueLyser II (Qiagen) for 2.5 min to homogenize the tissue. The tissue lysate was centrifuged at 14,000 rpm for 30 min at 4°C. The supernatant was used immediately for protein determination or stored at −80°C. The BCA (Thermo Fisher Scientific, Rockford, IL, USA) kit was used to quantify the total protein extracted from explant tissue in μg/ml as per the manufacturer’s instructions.

### Explant ELISAs

To assess angiogenic and inflammatory secretions from tumour conditioned media, a 17-plex ELISA kit separated across two plates was used (Meso Scale Diagnostics, USA). The multiplex ELISA determined the secreted levels of; IFN-γ, IL-10, IL-12p70, IL-13, IL-1β, IL-2, IL-4, IL-6, IL-8, TNF-α, bFGF, Flt-1, PlGF, Tie-2, VEGF-C, VEGF-D, and VEGF-A in tumour conditioned media. Assays were run as per the manufacturer’s recommendation; an overnight supernatant incubation protocol was used for the Pro-inflammatory Panel 1 with the Angiogenesis Panel 1 assay being run on the same day protocol. Tumour conditioned media were run undiluted on all assays as per previous optimization experiments. Secretion data for all factors were normalized to explant lysate protein content (extracted as described above) using a BCA protein assay kit (Thermo Fisher Scientific, Rockford, IL, USA).

### Correlation analysis

Following 72 h of culture, the secretion levels of 10 inflammatory and 7 angiogenic analytes were compared in *n* = 11 freshly isolated UM tumours treated with vehicle control or 1,4-dihydroxy quininib to determine if correlations existed between secretions in TCM and patients’ clinical parameters. Spearman correlation analysis was conducted using GraphPad Prism 7 software with a Spearman correlation value >0.6 considered a strong positive correlation and a Spearman correlation value < − 0.6 is considered a strong negative correlation.

### Uveal melanoma cell culture

UM cell lines derived from primary (Mel285) and metastatic (OMM2.5) UM were kindly provided by Dr. Martine Jager (Leiden, The Netherlands) (35–37). Cell lines were maintained at 37°C/5% CO_2_ in RPMI 1640 Medium (Gibco) supplemented with 10% FBS and 2% Penicillin/Streptomycin. All cell lines were routinely assessed for mycoplasma contamination.

### Western blotting in drug-treated uveal melanoma cells

UM cells were seeded at 2.5 × 10^5^ cells per well of a 6-well plate and left to adhere for 24 h. Cells were treated with DMSO or 20 μM of test compound for 1,2,5,8, or 24 h. Total protein was extracted from cells as described (18). PVDF membranes (MilliporeSigma, Burlington, MA, USA) were probed with primary antibodies (ERK: Santa Cruz [sc-514302], 1:1,000, phospho-ERK: Santa Cruz [sc-7383] 1:1,000, MITF: Proteintech [13092-1-AP] 1:1,000, Bcl-2: Proteintech [12789-1-AP] 1:1,000, COX-2: Proteintech [12375-1-AP] 1:500, Calpain-2: Abcam [ab39165] 1:1,000, β-actin: Santa Cruz [sc-47778], α-Tubulin: Santa Cruz, 1:1,000). Secondary antibodies were anti-mouse IgG HRP-linked (Cell Signaling [7076S] 1:1,000), or anti-rabbit IgG HRP-linked (Cell Signaling [7074S] 1:1,000). Signal was detected using enhanced chemiluminescence as per the manufacturer’s instructions (Pierce™ ECL Western Blotting Substrate, Thermo Fisher Scientific, Rockford, IL, USA).

### OMM2.5 cell line-derived orthotopic xenograft model

OMM2.5 metastatic uveal melanoma cells were cultured as described above and the OMM2.5 cell line-derived xenograft model was generated as previously described (18). Six to eight-week-old athymic Nude-Foxn1*^nu^* female mice (Envigo) were injected intrahepatically with 1 × 10^7^ OMM2.5 cells and monitored 3× weekly for tumour growth. Once tumours were grown, they were harvested and cut into tumour fragments of approximately 40 mm^3^. These solid tumours were implanted in the liver of 25 mice. Briefly, mice were anesthetized with a continuous flow of 1–3% isoflurane/oxygen mixture (2 L/min). After performing a median laparotomy, the tumour fragment was anchored with a Prolene 7-0 suture into a small pocket created in the anterior hepatic lobe (38). Five weeks after implantation, when homogeneous tumours are detected by palpation, mice were randomized and assigned to vehicle (5% DMSO, 25% PEG-400, and 75% H_2_O), 25 mg/kg of 1,4–dihydroxy quininib (prepared with 5% DMSO, 25% PEG-400, and 75% H_2_O), or 80 mg/kg dacarbazine (Medac, reconstituted with H_2_O). To reduce experimental variability, all cages were allocated mice receiving different treatments. Drug solutions were freshly prepared on the day of treatment administration. Mice were weighed immediately prior to drug administration to calculate the required dose for administration. All treatments were administered intraperitoneally every 3 days for 3 weeks (7 doses in total). Mice were housed in laminar flow rooms at a constant temperature (20–24°C) and humidity, with 5 animals per cage. Animals had free access to irradiation sterilized dry food and water during the study period. Mice behavior and weight were continuously monitored throughout the study.

### Immunohistochemical analysis of OMM2.5 cell line-derived xenograft tumour tissue

IHC for Ki-67 (Invitrogen—MA5-14520, 1:200), cleaved caspase 3 (Cell Signaling), and ATP5B (Sigma-Aldrich—HPA001520, 1:500) was performed on 4-μm FFPE sections produced from the xenograft tumours using commercial equipment and a DAB detection kit. Slides were counterstained with hematoxylin and mounted using DPX mountant (Sigma-Aldrich, St. Louis, MO, USA). Slides were scanned with an Aperio AT2 digital slide scanner (Leica Biosystem, Milton Keynes, United Kingdom) with a 20x lens and analyzed with imaging software (Aperio Image Scope version 11.2; Leica Biosystems, Inc., Buffalo Grove, IL, USA). Automated digital image analysis was performed using the Visiopharm Integrator System (Visiopharm, Hoersholm, Denmark). H-Score and percentage of positive cells were used as the image analysis output for cleaved caspase-3 and ATP5B expression. Percentage of positive cells was used as the image analysis output for Ki-67 expression. H-Score was calculated using the following formula: [1 × (% of weakly positive cells) + 2 × (% of moderately strong positive cells) + 3 × (% strong positive cells)].

### Analysis of the cancer genome atlas uveal melanoma dataset

Gene expression and clinical data from 80 primary UM included in The Cancer Genome Atlas (TCGA) were collected from the cBioPortal. RNA-seq data were downloaded in Fragments Per Kilobase of exon per million fragments Mapped (FPKM) and then converted to log2 scale. Survival analyses were performed with package “survminer,” R v3.5.0 (R Foundation for Statistical Computing, Vienna, Austria). Differences in *ATP5F1B* gene expression between recurrent and non-recurrent patients were tested by non-parametric Wilcoxon test. Associations between gene expression and prognosis were assessed by Cox proportional hazard regression models. Progression-Free Survival (PFS) and Overall Survival (OS) were used as end points. For categorization of the gene expression into “High” and “Low” categories, median values were used as cut-off. For combinatory analysis of gene expression with *ATP5F1B* or *BAP1* expression, samples were divided into four groups based on the combination of the gene expression (High-High; High-Low; Low-High; Low-Low). Chromosome 3 status was unavailable for 29 of the 80 patients from TCGA, leaving 51 patients to be included in the analysis of *ATP5F1B* expression and chromosome 3 status. Survival probabilities were plotted on a Kaplan–Meier curve, and a Log-rank test was used to compare the two groups. Disease-free survival is defined as time until metastatic recurrence. Overall survival is defined as death by any cause.

### Statistical analyses

Analysis of high expression of CysLT_1_ or CysLT_2_ associated with death from metastatic disease or overall survival was undertaken using Log-rank tests to compare survival across groups, and the Cox proportional hazards model. Survival time (years) was calculated from the date of first diagnosis until death, or study closure on 13 August 2020. All analyses were conducted using SPSS Statistics v.24 (IBM, Armonk, NY, USA). All other statistical analysis applied GraphPad Prism 7 software (GraphPad, San Diego, CA, USA). Specific statistical tests used are indicated in figure legends. For all statistical analyses, differences were considered statistically significant at *p* < 0.05.

## Results

### High expression of cysteinyl leukotriene receptor 1 is significantly associated with reduced survival in an independent primary uveal melanoma patient cohort

We previously reported that high CysLT_1_ expression significantly associates with reduced overall survival in a primary UM cohort from the UK (18). An appropriate approach to investigating potential prognostic biomarkers is to establish a hypothesis and cut-off value from a first patient cohort which is applied to analysis in an independent validation patient cohort (39). This is of particular importance in UM, wherein patient study numbers can be small due to the disease rarity. To validate the clinical relevance of CysLT receptors in UM, we analyzed CysLT_1_ and CysLT_2_ expression in a second TMA generated from primary UM of 94 consented patients treated at the Hospital Universitari de Bellvitge, Spain (Figure 1). Associated survival data was available for 68 patients: 39 males and 29 females with a median age of 61 years at primary management (range, 32–96). At the time of study end (13 August 2020), 33/68 UM patients were alive (48.5%), 31/68 had died from metastatic disease (45.6%) and 4/68 had died from other causes (5.9%). The median survival time was 5 years (range, 0.5–28 years) (Supplementary Figure 1D). The number of samples available for CysLT_2_ analysis was 48; 29 males and 19 females, with a median age of 62 years at primary management (range, 36–91 years). At the time of study end, 24/48 UM patients were alive (50%), 21/48 had died from metastatic disease (43.75%) and 3/48 had died from other causes (6.25%). The median survival time was 5 years (range, 0.8–28 years) (Supplementary Figure 1E). The chromosome 3 and BAP1 status of these patients was unavailable. Therefore, the relationship between CysLT_1_ expression and these genetic parameters could not be analyzed in this cohort. However, in our previously published UK UM patient cohort, there is no significant association between high CysLT_1_ expression and chromosome 3 status (18).

Manual analysis of both CysLT_1_ and CysLT_2_ expression was conducted using the median as a cut off, as established in our published UK cohort (18). In agreement, manual analysis demonstrated a significant relationship between high expression of CysLT_1_ and overall survival in UM patients (*p* = 0.015; HR 2.27; 95% CI 1.13–4.58) (Figure 1D). Additionally, this independent cohort revealed a significant relationship between high CysLT_1_ and death from metastatic disease (*p* = 0.021; HR 2.28; 95% CI 1.08–4.78) (Figure 1C). In Kaplan–Meier survival curves generated from scoring of 48 UM cases, immunohistochemical levels of CysLT_2_ did not demonstrate a significant association with overall survival (*p* = 0.69; HR 1.34; 95% CI 0.31–5.75) (Figure 1F) or survival from metastatic disease (*p* = 0.64; HR 1.39; 0.32–6.01) (Figure 1E), consistent with our previous data from UK patients (18).

Patient sex and age at primary management were the only additional clinical information accessible for patients in this cohort. Univariate analysis revealed that both sex (*p* = 0.048; HR 2.09; 95% CI 1.01–4.34) and age (*p* = 0.030; HR 1.03; 95% CI) were significantly associated with OS in this patient cohort. Multivariate analysis revealed that CysLT_1_ expression (*p* = 0.043; HR 2.23; 95% CI 1.07–4.65) and age (*p* = 0.018; HR 2.31; 95% CI 1.15–4.65) are independently associated with OS in this cohort.

### 1,4-dihydroxy quininib significantly alters the secretion of inflammatory factors in *ex vivo* explant tumours from primary uveal melanoma patients

**FIGURE 2:**
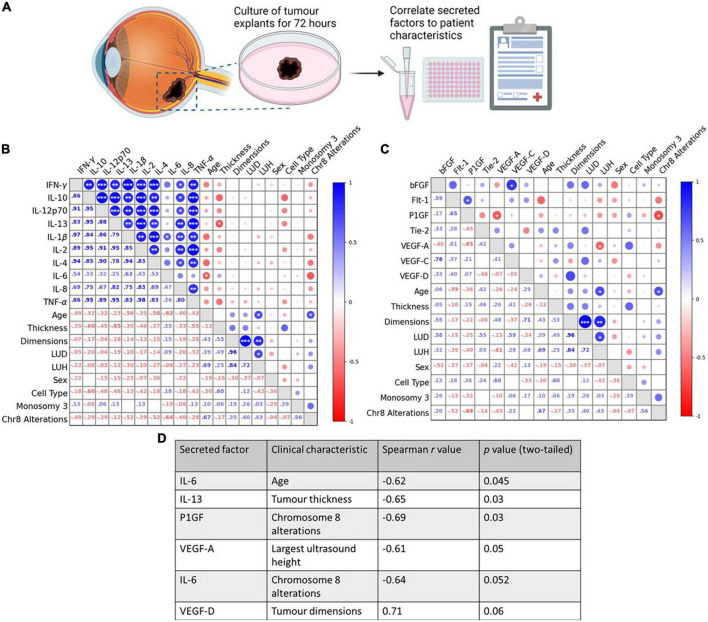
Tumour secretion of IL-6, IL-13, VEGF-A, and PlGF correlate with clinical characteristics in *ex vivo*, control-treated, primary UM patient tumours. **(A)** Schematic of the explant culture protocol with vehicle control. Following 72 h of culture, the secretion levels of 10 inflammatory and 7 angiogenic analytes were compared in *n* = 11 freshly isolated UM tumours treated with vehicle control, to determine if correlations existed between secretions in TCM and patients’ clinical parameters. **(B,D)** A significant negative correlation exists between IL-6 secretion and patient age, and IL-13 secretion and tumour thickness. **(C,D)** A significant negative correlation exists between VEGF-A secretion and largest ultrasound height, and PlGF secretion and chromosome 8 alterations. **(D)** Table shows all inflammatory and angiogenic secretions with a strong positive (Spearman correlation value > 0.6), or strong negative (Spearman correlation value < –0.6) correlation to a patient clinical characteristic (*n* = 11). Although not statistically significant (*p* > 0.05), IL-6 secretion shows a strong negative correlation with chromosome 8 alterations, and VEGF-D secretions shows a strong positive correlation with tumour dimensions. Spearman correlation value >0.6 is considered a strong positive correlation. Spearman correlation value <–0.6 is considered a strong negative correlation. The lower left triangle denotes Spearman correlation values for each comparison. Correlation analysis was conducted using GraphPad Prism 7 software. Correlations are visualized using a correlation plot generated by BioKit for Python. Conditioned media were collected and analyzed by ELISA (*n* = 11). Tumour tissue was snap-frozen for protein isolation. Secretion levels of inflammatory and angiogenic mediators were normalized to total protein content prior to statistical analysis. *p < 0.05; **p < 0.01; ***p < 0.001. LUD, largest ultrasound diameter; LUH, largest ultrasound height. Panel **(A)** created with BioRender.com.

**FIGURE 3:**
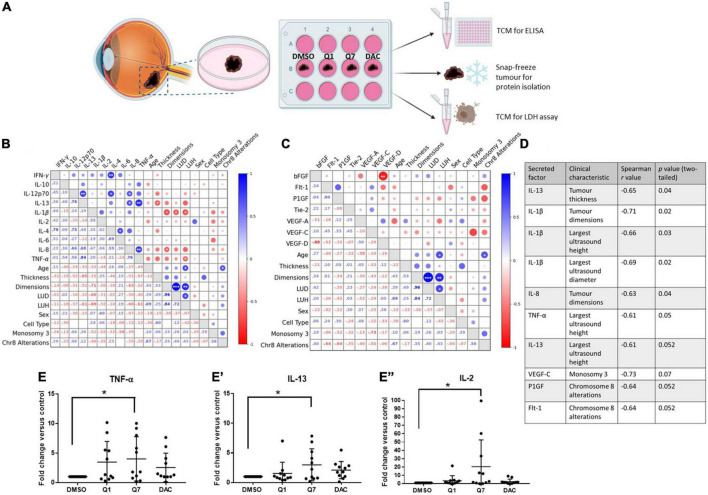
1,4-dihydroxy quininib significantly increases the secretion of IL-13, IL-2, and TNF-α in primary UM tumour explants vs. vehicle control. **(A)** Schematic of the explant culture protocol to compare drug effects. Each primary UM tumour was dissected into 4 fragments and grown in complete media with 20 μM quininib (Q1), 20 μM 1,4-dihydroxy quininib (Q7), 20 μM dacarbazine (DAC) or vehicle (DMSO) for 72 h, based on previously optimized explant culture experiments (29), before collection of the TCM. TCM was analyzed by ELISA, to detect the secretion of proinflammatory factors, and LDH assay to assess viability. Tumour tissue was snap-frozen for protein isolation. Secretion levels of inflammatory mediators were normalized to total protein content prior to statistical analysis. Additionally, secreted baseline levels of 10 inflammatory and 7 angiogenic analytes were compared in *n* = 11 patients treated with 1,4-dihydroxy quininib to determine if correlations existed between secretions in TCM and patients’ clinical parameters. **(B,D)** A significant negative correlation exists between IL-13 secretion and tumour thickness, IL-1β secretion and tumour dimensions, LUD, and LUH, IL-8 secretion and tumour dimensions, and TNF-α secretion and LUH. **(C,D)** No significant correlations were detected despite strong negative correlations between VEGF-C secretion and monosomy 3, and PlGF and Flt-1 secretion and chromosome 8 alterations. **(D)** Table shows all inflammatory and angiogenic secretions with a strong positive (Spearman correlation value > 0.6), or strong negative (Spearman correlation value < –0.6) correlation to a patient clinical characteristic (*n* = 11). Although not statistically significant (*p* > 0.05), IL-13 secretion shows a strong negative correlation with largest ultrasound height, VEGF-C secretion shows a strong negative correlation with monosomy 3, and PlGF and Flt-1 secretion show a strong negative correlation with chromosome 8 alterations. Spearman correlation value > 0.6 is considered a strong positive correlation. Spearman correlation value <–0.6 is considered a strong negative correlation. The lower left triangle denotes spearman correlation values for each comparison. Correlation analysis was conducted using GraphPad Prism 7 software. Correlations are visualized using a correlation plot generated by BioKit for Python. *p < 0.05; **p < 0.01; ***p < 0.001. LUD, largest ultrasound diameter; LUH, largest ultrasound height. In *ex vivo* primary UM tumour explants, 72-h treatment with 1,4-dihydroxy quininib significantly increases the secretion of TNF-α **(E)**, IL-13 **(E’)**, and IL-2 **(E”)** vs. vehicle control. Quininib or dacarbazine had no significant effect on the secretion of IL-13, IL-2, or TNF-α. Conditioned media were collected and analyzed by ELISA (*n* = 11). All secretions were normalized to total protein content and results calculated as fold change compared to vehicle control. Statistical analysis was performed by ANOVA with Dunnett’s *post-hoc* multiple comparison test. Error bars are mean ± S.D. *p < 0.05. Panel **(A)** created with BioRender.com.

To translate the UM disease relevance of CysLT_1_ into pharmacological relevance, we analyzed the effects of CysLT receptor antagonists on the cancer secretome of UM patient samples. 11 primary UM samples were obtained immediately post-enucleation from patients treated at the Royal Victoria Eye and Ear Hospital, Dublin. The primary UM samples were dissected into four pieces and tumour explants cultured in the presence of 20 μM quininib, 20 μM 1,4-dihydroxy quininib, 20 μM dacarbazine or vehicle control (DMSO) for 72 h before collection of the TCM (Figures 2A, 3A). ELISA quantified the levels of 10 inflammatory factors and 7 angiogenic factors. Throughout this manuscript dacarbazine is used as a clinical comparator against which the anti-cancer potential of quininib drugs is analyzed. All drug treatments, including dacarbazine, are compared to DMSO, the vehicle control.

We first determined if there was a correlation between the basal secretion of inflammatory or angiogenic factors from vehicle-treated primary UM samples with patient clinical characteristics, many of which are linked to prognosis (Supplementary Table 1). The secretion of factors was analyzed by ELISA and Spearman correlation analysis conducted to determine correlations between secreted factors and patient clinical characteristics. In vehicle-treated UM samples, a significant negative correlation was observed between IL-6 secretion and patient age, IL-13 secretion and tumour thickness, between VEGF-A secretion and largest ultrasound height (LUH), and between PlGF secretion and chromosome 8 alterations (Figures 2B–D).

We next sought to determine the correlation between the secretion of factors from tumours treated with 1,4-dihydroxy quininib and patient clinical characteristics (Figures 3B,C). In 1,4-dihydroxy quininib treated UM explants, a significant negative correlation was observed between IL-13 secretion and tumour thickness; IL-1β secretion and tumour dimensions (LUD and LUH); IL-8 secretion and tumour dimensions; and TNF-α secretion and LUH (Figures 3B–D). To analyze alterations in secreted factors in the TCM of drug treated explants, data were analyzed as fold change vs. vehicle control. In accordance, 1,4-dihydroxy quininib significantly increased the secretion of TNF-α (Figure 3E), IL-13 (Figure 3E’), and IL-2 (Figure 3E”) in primary UM samples vs. vehicle controls. Quininib and dacarbazine had no significant effect on the secretion of any of the factors analyzed (Supplementary Figures 2A–N).

### Cysteinyl leukotriene receptor 1 antagonists modulate phospho-ERK expression in uveal melanoma cell lines

**FIGURE 4:**
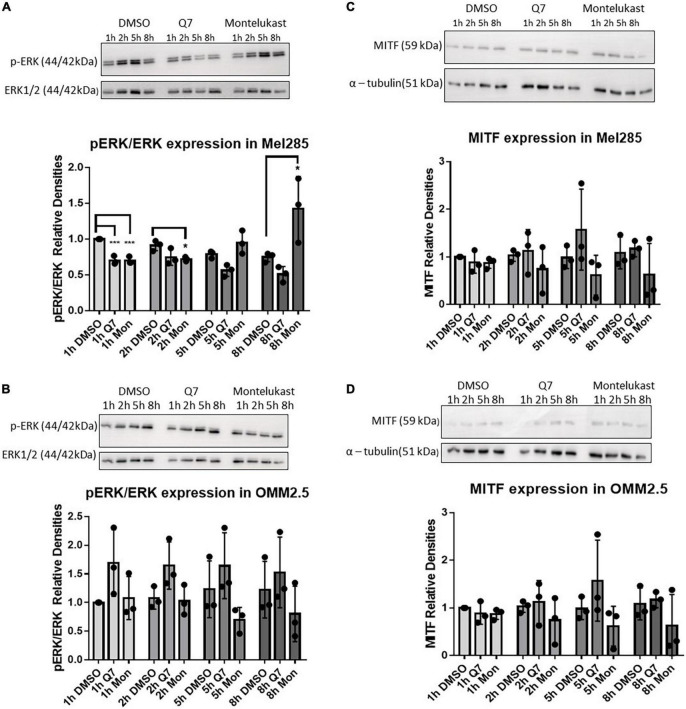
Treatment with CysLT_1_ antagonists alters expression of phospho-ERK in Mel285 cells. **(A–D)** Representative western blots and densitometry analysis. **(A)** Relative phospho-ERK expression was significantly reduced in Mel285 cells treated with 1,4-dihydroxy quininib or montelukast for 1 h and montelukast for 2 h. Relative phospho-ERK expression was significantly increased in Mel285 cells treated with montelukast for 8 h. **(B)** Relative phospho-ERK expression was not significantly altered by treatment with 1,4-dihydroxy quininib or montelukast in OMM2.5 cells at any time point analyzed. **(C,D)** MITF expression normalized to α-tubulin shows treatment with 1,4-dihydroxy quininib or montelukast does not significantly alter a 59 kDa isoform of MITF expression at any time point analyzed in Mel285 cells or OMM2.5 cells. Immunoblotting was conducted three separate times using protein lysates from three individual experiments. Differences in protein expression were assessed between drug treatments conducted for the same length of time. Statistical analysis was performed by ANOVA with Dunnett’s *post-hoc* multiple comparison test. Error bars are mean ± S.E. *p < 0.05; ***p < 0.001.

Before evaluating the *in vivo* therapeutic potential of CysLT_1_ antagonists, in a murine orthotopic xenograft model derived from OMM2.5 cells, we assessed if pathways linked to UM or CysLT_1_ signaling were altered in *in vitro* treated UM cell lines. We hypothesized that treating wildtype or mutated *GNAQ* UM cell lines with different CysLT_1_ antagonists could display differential changes in these pathways downstream of CysLT_1_. Less than 90% of UM possess mutations constitutively activating the MAPK/ERK pathway *via* alterations in Gα_q/11_ signaling (40). CysLTs enhance proliferation through ERK signaling (41, 42), and MAPK signaling is a cancer therapeutic target (43). Thus, we examined expression of phospho-ERK in Mel285 and OMM2.5 cells following treatment with CysLT_1_ antagonists for 1, 2, 5 and 8 h. As expected, in Mel285 cells, 1 h treatment with 1,4-dihydroxy quininib or montelukast significantly reduced relative phospho-ERK expression (Figure 4A). Treatment with montelukast for 2 h significantly reduced relative phospho-ERK expression, while surprisingly treatment with montelukast for 8 h significantly upregulated phospho-ERK (Figure 4A). In OMM2.5 cells, CysLT_1_ antagonists did not significantly alter relative phospho-ERK levels (Figure 4B). This is likely because these cells possess a Gα_q_ Q209P mutation which constitutively activates ERK (32).

Microphthalmia-associated transcription factor (MITF), a target of ERK phosphorylation (44), is dramatically upregulated in UM cell lines compared to normal uveal melanocytes and downregulation of MITF significantly inhibits proliferation and induces cell cycle arrest and apoptosis in UM cells (45–47). In Mel285 and OMM2.5 cells, a 59 kDa MITF antigen, likely the MITF-M isoform expressed predominantly in melanocytes (48) was not significantly altered by CysLT_1_ antagonists (Figures 4C,D). CysLT mediated cell survival and proliferation is linked to COX-2 and Bcl-2, both linked to the MAPK pathway (49). 1,4-dihydroxy quininib reduced calpain-2 expression in HMEC-1 cells and in an *in vivo* xenograft model of colorectal cancer (29, 34). However, in Mel285 and OMM2.5 cells treated for 24 h with CysLT_1_ antagonists, expression of COX-2 (Supplementary Figures 3A,B), Bcl-2 (Supplementary Figures 3C,D), or Calpain-2 (Supplementary Figures 3E,F) was not significantly altered. In summary, treatment with CysLT_1_ antagonists significantly alters phospho-ERK expression in a time, and cell line mutation dependent manner in UM cells *in vitro*.

### 1,4-dihydroxy quininb significantly reduces expression of ATP5B in a cell line-derived metastatic uveal melanoma xenograft model

**FIGURE 5:**
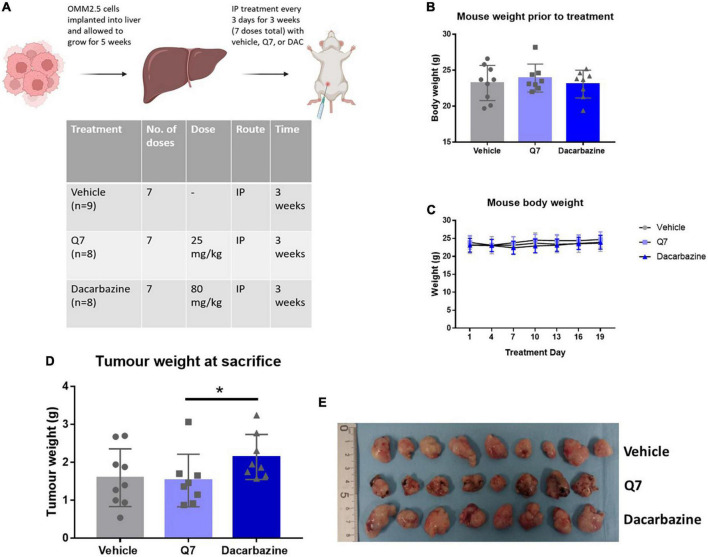
Treatment with 1,4-dihydroxy quininib or dacarbazine did not significantly reduce tumour weight in an OMM2.5 cell line-derived xenograft model of metastatic UM. **(A)** OMM2.5 cell line-derived liver tumour fragments were implanted intrahepatically in mice and allowed grow for 5 weeks. Mice were randomized to treatment with vehicle, 1,4-dihydroxy quininib (Q7), or dacarbazine. All animals received seven doses intraperitoneally over a 3-week period. **(B)** Prior to study commencement, all mice with palpable tumours were randomized to vehicle (*n* = 9), 1,4-dihydroxy quininib (Q7) (*n* = 8), or dacarbazine (*n* = 8). There were no significant differences in the weight of animals across the three different treatment groups before the study began. Body weight mean ± SD was 23.2 g ± 2.4 for vehicle, 23.9 g ± 1.9 for Q7, and 23.1 g ± 1.9 for dacarbazine. Statistical analysis was performed by a Kruskal-Wallis test. **(C)** Body weights were monitored every 3 days during the study period. No statistically significant differences in body weight were observed between treatment groups throughout the study. Statistical analysis was performed by a two-way repeated measures ANOVA. Error bars are mean ± SD. **(E)** Following necropsy, liver tumours were resected and arranged by treatment group. Individual tumours were weighed and the average weight of tumours from each group was compared. **(D)** Treatment with 1,4-dihydroxy quininib (Q7) or dacarbazine did not result in significant changes in tumour weight vs. vehicle control (*p* = 0.81 and *p* = 0.14, respectively). **(D)** In animals treated with dacarbazine, tumour weight was significantly higher (*p* = 0.023) than those treated with 1,4-dihydroxy quininib. Tumour weight mean ± SD was 1.59 g ± 0.76 for vehicle treated mice, 1.52 g ± 0.69 for 1,4-dihydroxy quininib treated mice, and 2.14 g ± 0.59 for dacarbazine treated mice. Statistical analysis was performed using a Mann-Whitney *U* test to compare differences between two groups. Error bars are mean ± S.D. *p < 0.05. Panel **(A)** created with BioRender.com.

An OMM2.5 cell line-derived, rodent xenograft model of MUM was established in the liver as previously described (18). Based on efficacy in the primary UM patient explants, 1,4-dihydroxy quininib was chosen as the CysLT_1_ antagonist to therapeutically evaluate. Five weeks after implantation, mice with palpable, homogenous, metastatic UM tumours were randomly assigned to treatment with vehicle control, 1,4-dihydroxy quininib or dacarbazine (Figure 5A). Each treatment was administered by intraperitoneal injection, every 3 days for a period of 3 weeks, with each animal receiving seven doses in total. There were no significant differences in the weight of animals across the different treatment groups before the study began (Figure 5B) or throughout the treatment regimen (Figure 5C). Tumours grew in the livers of all mice included in the study (Supplementary Figure 4). Following sacrifice, all primary liver tumours were weighed to determine differences between treatment groups. The weight of tumours from mice treated with dacarbazine, a chemotherapeutic drug administered to UM patients, was significantly larger than those treated with 1,4-dihydroxy quininib (*p* = 0.023) (Figures 5D,E). However, there was no significant difference in weight between either drug intervention or vehicle control (Figures 5D,E). Within all three treatment groups, metastases to the peritoneal wall, mesentery or the diaphragm were observed. Indeed, cases of peritoneal metastases are reported in UM patients, secondary to their liver metastases (50, 51). In the vehicle group, the number of animals with metastases to the peritoneal wall was significantly higher than in the 1,4-dihydroxy quininib or dacarbazine groups (*p* < 0.0001) (Supplementary Figure 5A). The number of animals with metastases to the mesentery was significantly lower in the dacarbazine treated group compared to both the vehicle and 1,4-dihydroxy quininib treated groups (*p* < 0.0001) (Supplementary Figure 5B). There were no significant differences in the number of animals with metastases in the diaphragm across all three treatment groups (Supplementary Figure 5C). Metastases to the peritoneal wall, mesentery, and diaphragm can be grouped as peritoneal metastases. The percentage of animals presenting with metastases at all sites was significantly lower in the dacarbazine treated group vs. both the vehicle and 1,4-dihydroxy quininib treated groups (*p* < 0.0001) (Supplementary Figure 5D). The percentage of animals presenting with metastases in more than one site within the peritoneum was significantly higher in the vehicle treated group compared to both the 1,4-dihydroxy quininib and dacarbazine treated groups (*p* < 0.0001) (Supplementary Figure 5E).

**FIGURE 6:**
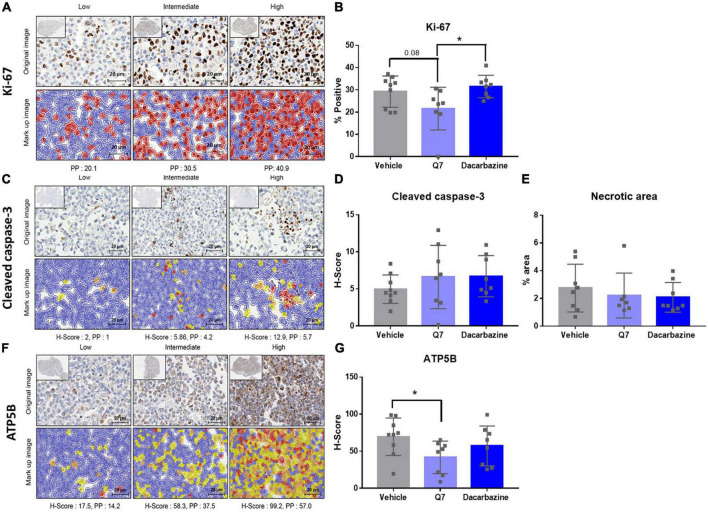
1,4-dihydroxy quininib significantly reduces expression of ATP5B in a UM cell line-derived orthotopic xenograft model of metastatic uveal melanoma. **(A–G)** Representative digital pathology images and quantification graphs. **(A)** Representative sections, and associated marked up images, designated with a score of *Low*, *Intermediate* or *High* expression of Ki-67. Cells positive for Ki-67 staining are indicated in red and cells negative for Ki-67 staining are indicated in blue in the mark up images. **(B)** Treatment with 1,4-dihydroxy quininib (Q7) does not significantly alter the percentage of cells staining positive for Ki-67 vs. vehicle control (*p* = 0.08). Treatment with 1,4-dihydroxy quininib significantly (*p* = 0.02) alters the percentage of cells staining positive for Ki-67 vs. dacarbazine. There is no significant difference between vehicle and dacarbazine treated groups (*p* = 0.45). **(C)** Representative sections, and associated mark up images, designated with a score of *Low*, *Intermediate*, or *High* expression of cleaved caspase-3 following digital pathology analysis. Blue indicates negative staining, yellow indicates H-Score of +1, orange indicates H-score of +2, red indicates H-Score of +3. **(D)** As assessed by H-Score, 1,4-dihydroxy quininib (Q7) and dacarbazine do not significantly alter cleaved-caspase 3 expression vs. vehicle (*p* = 0.31 and *p* = 0.15, respectively). **(E)** The percentage of necrotic area was quantified using H&E tumour sections from each treatment group. There was no significant difference in the necrotic area between any treatment groups. **(F)** Representative sections, and associated mark up images, designated with a score of *Low*, *Intermediate*, or *High* expression of ATP5B. Blue indicates negative staining, yellow indicates H-Score of +1, orange indicates H-score of +2, red indicates H-Score of +3. **(G)** As assessed by H-Score, 1,4-dihydroxy quininib (Q7) significantly (*p* = 0.03) reduces the expression of ATP5B vs. vehicle control. Dacarbazine does not significantly alter ATP5B expression vs. vehicle (*p* = 0.35). Statistical analysis was performed using an unpaired two-tailed *t*-test. Error bars are mean ± SD. *p < 0.05.

Tumours from animals treated with 1,4-dihydroxy quininib appeared smaller overall (Figure 5E). Therefore, to evaluate cellular efficacy readouts, we analyzed all xenograft tumours by IHC and digital pathology. Ki-67, a marker of cell proliferation, is strongly associated with tumour cell proliferation and growth and correlates with metastasis and tumour staging (52). OMM2.5 cells express high levels of Ki-67 compared to other UM cells (53). Despite a decrease of 7.5%, the percentage of Ki-67 positive cells was not significantly reduced (*p* = 0.08) following 1,4-dihydroxy quininib treatment compared to vehicle control (Figures 6A,B). The percentage of Ki-67 positive cells was significantly lower (9.9% decrease) in 1,4-dihydroxy quininib treated mice vs. those treated with dacarbazine (Figure 6B) (*p* = 0.02).

The anti-cancer effects of CysLT_1_ antagonist montelukast in prostate, testicular, and breast cancer cells is attributed to the induction of apoptosis (16, 22, 23). As assessed by digital pathology, there was no significant difference in cleaved caspase-3 expression between any of the treatment groups (vehicle average H-Score; 4.96, 1,4-dihydroxy quininib average H-Score; 6.60, dacarbazine average H-Score; 6.72) (Figures 6C, D). In addition, there was no significant difference in the percentage of necrotic area between treatment groups (Figure 6E).

Finally, we previously showed CysLT_1_ antagonists to significantly reduce oxidative phosphorylation in primary and metastatic UM cells *in vitro* (18). Therefore, we examined the expression of ATP5B, a marker of oxidative phosphorylation, in the post-mortem xenograft tumour tissues (Figure 6F). When assessed by H-Score, 1,4-dihydroxy quininib significantly reduces ATP5B levels vs. vehicle (*p* = 0.03) (Figure 6G). Treatment with dacarbazine had no significant effect on ATP5B levels vs. vehicle (*p* = 0.35) (Figure 6G).

### High *ATP5F1B* expression is associated with poor outcomes in primary uveal melanoma

**FIGURE 7:**
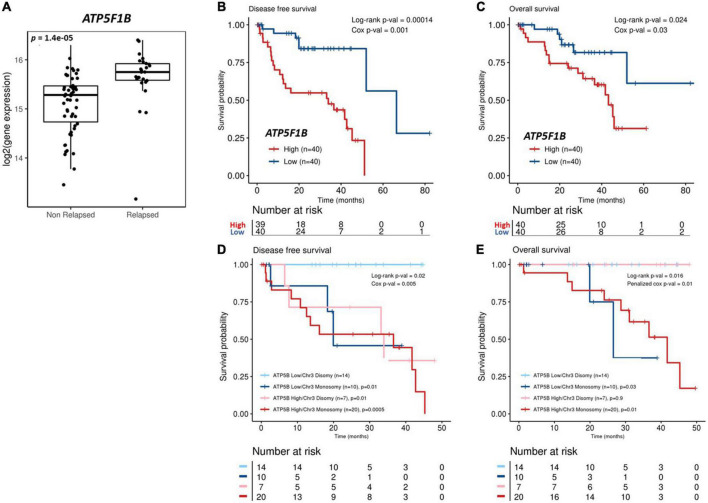
Analysis of *ATP5F1B* expression and UM patient outcomes from The Cancer Genome Atlas (TCGA). **(A)** Patients from the TCGA-UM dataset were categorized into those that received a diagnosis of primary UM, but their disease had not progressed (non-relapsed) vs. those who had developed recurrent disease (relapsed). *ATP5F1B* expression in primary UM is significantly higher (*p* < 0.001) in UM patients presenting with relapsed disease. Statistical analysis was carried out using a non-parametric Wilcoxon test. Kaplan–Meier survival curves demonstrate a statistically significant relationship between high (red) *ATP5F1B* expression and disease-free survival **(B)** (*n* = 80; Log-rank; *p* = 0.00014) or overall survival **(C)** (*n* = 80; Log-rank; *p* = 0.024) in UM patients. Low *ATP5F1B* expression is shown in blue **(B,C)**. Monosomy 3 is a strong prognostic indicator of overall survival, irrespective of *ATP5F1B* expression **(E)**. There is a significant difference in disease free survival in disomy 3 patients combined with low *ATP5F1B* expression compared to those with monosomy 3 and low *ATP5F1B* (*p* = 0.01), those with disomy 3 and high *ATP5F1B* expression (*p* = 0.01), and those with monosomy 3 and high *ATP5F1B* expression (*p* = 0.0005) **(D)**. Both Log-rank *p*-values and Cox *p*-values were calculated and are displayed.

Oxidative phosphorylation is upregulated in invasive melanoma (54) and the metabolic switch of some melanomas to oxidative phosphorylation is linked to resistance of MAPK pathway inhibitors (55). Similarly, ATP5B participates in carcinogenesis and is associated with poor outcomes in several malignancies (56–58). Thus, to further interrogate the significance of ATP5B expression in primary UM, we analyzed gene expression from 80 primary UM from The Cancer Genome Atlas. *ATP5F1B* transcript expression is significantly higher (*p* < 0.0001) in patients with UM with recurrent disease (Figure 7A). Additionally, high expression of *ATP5F1B* in primary UM is significantly associated with reduced disease-free survival (*p* < 0.0001) (Figure 7B) and reduced overall survival (*p* = 0.024) (Figure 7C). Given that chromosome 3 status is currently the principal determinant of metastatic risk in UM, we stratified that data based whether patients presented with monosomy or disomy 3 and high or low expression of *ATP5F1B*. As anticipated, monosomy 3 is indeed a strong prognostic indicator of overall survival, irrespective of *ATP5F1B* expression (Figure 7E). However, there is a significant difference in disease free survival in disomy 3 patients combined with low *ATP5F1B* expression compared to those with monosomy 3 and low *ATP5F1B* (*p* = 0.01), those with disomy 3 and high *ATP5F1B* expression (*p* = 0.01), and compared to those with monosomy 3 and high *ATP5F1B* expression (*p* = 0.0005) (Figure 7D). This suggests that patients with disomy 3 and low *ATP5F1B* expression have a reduced risk of metastatic disease vs. patients with disomy 3 and high *ATP5F1B* (Figure 7D). We next assessed *BAP1* expression in combination with *ATP5F1B* expression. Here, we see a significant difference in disease free survival between patients with high *BAP1* expression and low *ATP5F1B* expression vs. all other groups, including those with high *BAP1* expression and high *ATP5F1B* expression (*p* = 0.02) (Supplementary Figure 6A). Interestingly, there is a significant difference in disease-free survival between those with low *BAP1* and low *ATP5F1B* vs. those with low *BAP1* and high *ATP5F1B* expression (*p* < 0.001) (Supplementary Figure 6A). In terms of overall survival, we see that *BAP1* expression is a strong prognostic indicator (Supplementary Figure 6B). However, we do see a difference, although not statistically significant (*p* = 0.07), between patients with high *BAP1* and low *ATP5F1B* expression and those with high *BAP1* and high *ATP5F1B* expression. These findings suggest that patients with low *ATP5F1B* and high *BAP1* expression have an advantage in terms of and disease-free survival vs. those with high *BAP1* and high *ATP5F1B* expression. Altogether, this shows that recurrent UM is linked with higher levels of *ATP5F1B* expression and that *in vivo* the CysLT_1_ antagonist 1,4-dihydroxy quininib can significantly reduce ATP5B protein levels in an orthotopic xenograft model of metastatic UM.

## Discussion

A diagnosis of metastatic UM remains associated with a poor patient prognosis. Herein, we further evaluated the clinical potential of CysLT receptors, both as biomarkers and therapeutic targets in UM. We validated that high CysLT_1_ expression correlates with poor survival in an independent primary UM patient cohort, demonstrating robustness and reproducibility across different patient cohorts. Our data strengthens the significance of cysteinyl leukotriene signaling in UM and suggests that CysLT_1_ could contribute to a biomarker panel to identify patients likely to experience a poor outcome. It will be interesting to assess if CysLT_1_ expression is a predictor of treatment response, to CysLT antagonists or other targeted or immunotherapies, in patients with UM.

We previously reported the anti-cancer potential of quininib CysLT_1_ antagonists in primary and metastatic UM cells *in vitro* (18). The same significant effects were not produced by treating UM cells with montelukast (18). Owing to this, and the questions surrounding the selectivity (59) and side effects (60) associated with montelukast, this work focuses exclusively on the progression of quininib CysLT_1_ antagonists. Although 1,4-dihydroxy quininib can inhibit VEGFR-2 and VEGFR-3 kinase activity at higher concentrations (34), there are several lines of evidence supporting that the most relevant target of 1,4-dihydroxy quininib is likely CysLT_1_. Firstly, 1,4-dihydroxy quininib can produce anti-angiogenic effects independent of VEGF, supporting a distinct mechanism of action of this drug (34). In addition, 1,4-dihydroxy quininib does not phenocopy Bevacizumab, a drug known to specifically block the VEGF-VEGFR signaling pathway (28). To our knowledge, this study is the first to apply advanced, *ex vivo* patient and *in vivo* xenograft preclinical models to investigate the therapeutic potential of CysLT_1_ antagonism in UM. *Ex vivo* culture of patient tumours better recapitulates the tumour microenvironment by incorporating heterogenous tumour cells along with associated stroma and tumour infiltrating leukocytes (61). They also confer the opportunity to analyze personalized patient responses to drug treatments.

Our previous work identified quininib CysLT_1_ antagonists to significantly alter the secretion of cancer-associated inflammatory and angiogenic factors from UM cell lines (18). Using fresh primary UM tumour samples donated by patients undergoing eye enucleation surgery, we determined here if similar effects were observed in *ex vivo* tumours. We analyzed correlations between secretion of inflammatory and angiogenic factors and patient characteristics in primary UM samples treated with vehicle or 1,4-dihydroxy quininib. Interestingly, all significant correlations negatively correlate with a patient characteristic, many of which are indicators of poor prognosis (20, 62). For example, high secretion of IL-13 negatively correlates with tumour thickness in both vehicle and 1,4-dihydroxy quininib treated tumours. Tumour size (including tumour thickness and the largest basal diameter) is one of the most important prognostic characteristics of UM (62). Notably, high IL-13 levels in the vitreous of UM patients is associated with greater survival (63).

In control-treated tumour specimens, there were strong negative correlations between IL-6 secretion and age, IL-6 secretion and chromosome 8 alterations, and VEGF-A secretion and largest ultrasound height. A strong positive correlation was detected between VEGF-D secretion and tumour dimensions. None of the above correlations were maintained when examining the secretions from 1,4-dihydroxy quininib treated tumours. Given the patient samples remain constant, the level of secretion of IL-6, VEGF-A, and VEGF-D must be altered in tumours treated with 1,4-dihydroxy quininib. The negative correlations between IL-13 secretion and tumour thickness, and PlGF secretion and chromosome 8 alterations were maintained in both control and 1,4-dihydroxy quininib treated tumours. In addition, a negative correlation between IL-13 secretion and largest ultrasound height was detected in 1,4-dihydroxy quininib treated tumours. This, and the significant negative correlation detected between TNF-α secretion and largest ultrasound height, is not surprising given that both IL-13 and TNF-α secretion are significantly upregulated following 1,4-dihydroxy quininib treatment. In agreement with our findings, the secretion of TNF-α in the vitreous of patients with UM negatively correlates with largest basal diameter (63). In addition, significant negative correlations were detected between IL-1β secretion and tumour dimensions, LUH and LUD, and IL-8 and tumour dimensions in 1,4-dihydroxy quininib treated samples. Again, this suggests that secretion of IL-1β and IL-8 differs between control and 1,4-dihydroxy quininib treated samples as the clinical characteristics of the patients are unchanged.

In primary UM samples, treatment with quininib or 1,4-dihydroxy quininib did not significantly alter the secretion of any angiogenic factors analyzed. However, 1,4-dihydroxy quininib significantly upregulated the secretion of inflammatory factors IL-13 (2.3-fold), IL-2 (2.8-fold), and TNF-α (1.8-fold) vs. vehicle control in primary UM patient samples. Interestingly, the secretion of factors following drug treatment differed quite substantially to those detected using homogenous UM cell lines (18). Following 24 h of treatment in Mel285 cells, quininib significantly decreased IL-6 and IL-2 secretion (18). While in OMM2.5 cells, quininib significantly increased the secretion of IL-13, IL-10, IL-6, IL-1β, IL-8, IL-12p70, IL-2, and TNF-α following 24 h of treatment (18). Despite significant effects mediated by quininib on the secretion of inflammatory mediators in UM cell lines (18), quininib did not significantly alter the secretion of any factors analyzed in primary UM samples. The milder effects of the quininib drugs, in terms of the number of factors altered, is not surprising given the heterogeneity of cells within the *ex vivo* tumours and that each tumour sample arises from a unique UM patient. Indeed, that these 3 factors remain significantly altered by 1,4-dihydroxy quininib despite the large biological sample variation, highlights the robustness of this change across patient samples.

Immunotherapy has dramatically altered patient outcomes in cutaneous melanoma (64), yet the same benefits have not been realized in UM. The low mutational burden, the immune-privileged environment of the eye, and the immune-modulatory microenvironment of the liver are all linked to immune suppression in UM (65–68). Strategies to enhance the immunogenicity of UM, and therefore potential response to immunotherapies are desirable. Therefore, enhanced secretion of certain inflammatory cytokines, such as those secreted following treatment with 1,4-dihydroxy quininib, may be beneficial in the treatment of UM, particularly in the context of combination therapy approaches.

Before progressing to an *in vivo* study with an OMM2.5 orthotopic xenograft model, we investigated if candidate signaling pathways were altered by CysLT_1_ drugs *in vitro*. Activation of the MAPK pathway is extremely common in UM and CysLT_1_ antagonists downregulate phospho-ERK expression in cancer and non-cancer cell lines (30, 31). Our results suggest CysLT_1_ antagonism does transiently and mildly alter MAPK signaling in G_aq_ wildtype Mel285 primary UM cells, but not in G_aq_ Q209P metastatic UM cells. The insignificant effects of 1,4-dihydroxy quininib on ERK in OMM2.5 cells is not surprising as these cells contain a G_αq_ Q209P mutation which constitutively activates ERK. None of the CysLT receptor antagonists tested significantly altered the levels of MITF, Bcl-2, calpain-2 or Cox-2 under the conditions assessed. Effects of CysLT receptor agonists or antagonists on COX-2 and Bcl-2 expression is cell line-dependent, which may explain the absence of changes observed in UM cells. Montelukast significantly effects expression of COX-2 and Bcl-2 in A549 lung cancer cells, while no significant effect was detected in CL-15 lung cancer cells, despite montelukast inhibiting proliferation in both cell lines (21). 1,4-dihydroxy quininib significantly reduces calpain-2 expression in HMEC-1 cells (34) and in a xenograft model of CRC assessed by IHC (29). Calpains are strongly involved in angiogenesis (69). Downregulation of Calpain-2 in HMEC-1 cells by 1,4-dihydroxy quininib corresponds with a decrease in VEGF-A secretion (34). In contrast, quininib and 1,4-dihydroxy quininib significantly upregulate secretion of VEGF-A in the UM cell lines used here (18), which may explain the absence of changes in calpain-2 expression here. Despite the limited molecular changes observed by Western blotting, we proceeded to the MUM xenograft model, as the CysLT_1_ antagonists reduce proliferation and cellular metabolism of OMM2.5 cells *in vitro* and modulate the cancer secretome of patient UM explants *ex vivo*. Based on the significant effects in our primary UM explant culture model, 1,4-dihydroxy quininib was chosen for further study in our orthotopic xenograft model compared to dacarbazine. Interestingly, dacarbazine significantly increased tumour weight and expression of Ki-67 vs. 1,4-dihydroxy quininib treated mice. This finding corroborates our *in vitro* data (18), UM explant data, and UM clinical trials wherein dacarbazine has negligible therapeutic effects (70). In relation to 1,4-dihydroxy quininib, following 3 weeks of well-tolerated treatment, no significant reductions in tumour weight were observed compared to vehicle control. However, the intrahepatic tumour location prevented continuous monitoring of tumour size over time. Therefore, dynamic changes in tumour size may have occurred during earlier phases of the treatment regimen. Indeed, the lack of reduction in tumour weight could be considered a negative finding, however, it is important to note that tebentausp can produce clinically beneficial outcomes for patients, even in the absence of a radiographically significant decrease in tumour size (8). Thus, we investigated if 1,4-dihydroxy quininib induced relevant molecular changes in the murine xenografts. Despite a 7.5% reduction, 1,4-dihydroxy quininib did not significantly (*p* = 0.08) reduce Ki-67 expression vs. vehicle control. Likewise, the expression of cleaved caspase-3 was not significantly altered. These results are in agreement with previous CRC subcutaneous xenograft models, wherein 1,4-dihydroxy quininib did not significantly alter Ki-67 (*p* = 0.09) or cleaved caspase-3 (*p* = 0.10) expression (29).

Importantly, 1,4-dihydroxy quininib significantly reduced expression of ATP5B, a protein marker of oxidative phosphorylation vs. vehicle in the orthotopic MUM xenograft model. To our knowledge, this is the first study to show that metabolism can be pharmacologically altered *in vivo*, in an orthotopic xenograft model of metastatic UM. This agrees with published *in vitro* data in primary and metastatic UM cells (18). ATP5B, a subunit of ATP synthase, promotes carcinogenesis *via* increased proliferation, migration, and invasion (56) and increased ATP5B expression is associated with poor outcomes in several malignancies (56–58). ATP, which can be secreted into the extracellular environment (71), signals through activation of purinergic P2 receptors (P2R) (72). Interesting complexity arises as CysLT receptors are the closest homologs of P2R (73), heterodimerization of these receptor classes is reported (74), and the CysLT receptor agonist LTE_4_ can also signal through P2Y_12_ (75, 76). In accordance, CysLT antagonists may also antagonize P2Y signaling independent of CysLT_1_ antagonism (77, 78), or by parallel antagonism of both CysLT_1_ and P2Y_12_ (31). It will be interesting to unravel if this mechanism contributes to how 1,4-dihydroxy quininib reduces ATP5B expression and whether antagonism of P2Y signaling contributes to the *ex vivo* and *in vivo* effects in UM models. The liver is a difficult organ to target pharmacologically due to many compounds being cleared by first-pass metabolism (79), this adds further significance to the ability of 1,4-dihydroxy quininib to mediate molecular changes *in vivo*, in a cancer with a notoriously poor prognosis. Our data provides proof-of-concept that 1,4-dihydroxy quininib can be safely and successfully administered intraperitoneally and can mediate molecular changes in liver tumours.

Increased mRNA and protein expression of ATP5B is reported in breast cancer and high ATP5B expression is associated with a worse prognosis (80). Herein, we show for the first time that *ATP5F1B* expression is significantly associated with a poor prognosis in primary UM. *ATP5F1B* expression is significantly higher in UM patients with relapsed disease. In keeping with this finding, high expression of *ATP5F1B* is significantly associated with reduced disease-free and overall survival in primary UM. When patients are stratified based on their chromosome 3 status and *ATP5F1B* expression levels, those with disomy 3 and low *ATP5F1B* expression have significantly better disease-free survival compared to all other groups. This combination may distinguish disomy 3 patients that are less likely to develop metastatic disease. Modulation of oxidative phosphorylation can control proliferation of tumour cells (81), which may explain the effect of CysLT_1_ antagonists on UM cell survival and proliferation (18). Comparing 31 tumour types, UM ranked amongst the tumours with the highest oxidative phosphorylation signature (82) and targeting of MEK and CDK4/6 in UM leads to adaptive upregulation of oxidative phosphorylation (83). Inhibition of oxidative phosphorylation can enhance the efficacy of targeted therapy in *in vitro* and *in vivo* models of UM (83). Our data further highlights that targeting this process in UM may enhance treatment efficacy and indicates the potential use of CysLT_1_ antagonists to inhibit OXPHOS in a combinatorial treatment approach with alternative immune or traditional targeted therapies. UM does not exhibit a classical Warburg effect (84). Onken et al. (84) report that that oncogenic G_q/11_ signaling in UM cell lines promotes both glycolytic activity and mitochondrial respiration (84). Additionally, metabolic heterogeneity in *BAP1* mutant vs. *BAP1* wild-type UM was recently described based on oxidative phosphorylation gene expression profiles (85). Thus, there may be patient cohorts that most benefit from CysLT_1_ antagonist drugs, that can effectively inhibit oxidative phosphorylation in UM.

Drugs like 1,4-dihydroxy quininib that target multiple cancer hallmarks, such as inflammation and metabolism, are favorable and less likely to be affected by treatment resistance. Here, we propose a conceptual mechanism linking all observed effects of 1,4-dihydroxy quininib on UM tumour biology. In the simple monocultures of primary and metastatic UM cell lines, 1,4-dihydroxy quininib functionally reduces oxidative phosphorylation (18). In agreement, in the metastatic UM cell-line derived xenograft model, 1,4-dihydroxy quininib reduces the expression of ATP5B, a marker of oxidative phosphorylation. While CysLTs are predominantly linked to inflammation and angiogenesis, they also regulate metabolism *via* translocation of β-catenin to the mitochondria and nucleus (27). Therefore, conceptually, 1,4-dihydroxy quininib mechanistically reduces OXPHOS by antagonizing CysLT_1_ signaling in UM cell lines and modulating PI3K and β-catenin activity.

Following treatment with 1,4-dihydroxy quininib, significant increases in secretion of IL-13, IL-2 and TNF-α are observed in the primary tumour explants, but not in the UM cell lines (18). This suggests a key role for the 3D tumour architecture or other cells present in the tumour microenvironment (e.g., dendritic or NK cells). IL-13 is produced by NK cells which prevent metastases or to kill tumour cells in the circulation before reaching the liver in *in vivo* UM models (86, 87). Increased IL-13 may promote NK cell activity which may decrease the metastatic potential of UM cells (63). Similarly, the role of IL-2 in the proliferation and expansion of NK cells is well-established (88) and *in vivo*, the anti-tumour activity of activated NK cells depends on the continuous availability of IL-2 (89). NK cells also produce TNF-α (88) and NK cells exert anti-tumour functions by inducing apoptosis through TNF-α (90). Dendritic cells play a predominant role in NK cell activation and similarly, IL-2 activated NK cells induce immature DC activation (91). TNF-α is also involved in bidirectional cross-talk between NK cells and DCs (91). The increased secretion of inflammatory cytokines likely also impacts metabolism in the TME. TNF-α can inhibit oxidative phosphorylation in hepatocytes, which can ultimately lead to cell death caused by energy depletion (92). In turn, an inhibition or decrease in oxidative phosphorylation can be linked to dendritic cell activation (93) and can render cancer cells more susceptible to NK cell mediated cytotoxicity. Indeed, increased vitreous concentrations of IL-13 in eyes with UM correlates with statistically improved overall survival (63). Similarly, the beneficial effects of tebentafusp in UM are linked to the secretion of cytokines including TNF-α and IL-2 (2).

This study advances translation of the known effects of the CysLT_1_ antagonist, 1,4-dihydroxy quininib, into clinically relevant *in vivo* and *ex vivo* models of primary and metastatic UM. UM patient samples are extremely rare and precious research samples. While this may present limitations on the scale of the research that can be conducted, the vastly superior physiological and clinical relevance of these models compared to results obtained using immortalized cell lines makes the arising data more relevant and significant. Further work on how this may improve treatment responsiveness, in combination with existing or experimental therapies, in UM patients is needed. Similarly, future work should focus on identifying specific subsets of patients that may benefit from such interventions that can effectively manipulate the tumour microenvironment.

## Data availability statement

The raw data supporting the conclusions of this article will be made available by the authors, without undue reservation.

## Ethics statement

The studies involving human participants were reviewed and approved by the Royal Victoria Eye and Ear Hospital Research Ethics Committee and Hospital de Bellvitge Clinical Research Ethics Committee. The patients/participants provided their written informed consent to participate in this study. This animal study was reviewed and approved by the Ethical Committee of Animal Experimentation of the Parc Científic de Barcelona.

## Author contributions

KS: conceptualization, methodology, validation, formal analysis, investigation, writing—original draft, writing—review and editing, visualization, project administration, and funding acquisition. RB: methodology, formal analysis, investigation, and writing—review and editing. KFS: formal analysis, investigation, and writing—review and editing. CJ: investigation and visualization. SG-M: formal analysis, investigation, and visualization. AR: methodology and intellectual input. FO’C: intellectual input. JP and VO’N: resources. NH: resources and intellectual input. SC: investigation, resources, writing—review and editing, and intellectual input. JO’S: conceptualization, methodology, writing—review and editing, and intellectual input. WG and AV: methodology, resources, writing—review and editing, and intellectual input. BK: conceptualization, methodology, writing—original draft, writing—review and editing, supervision, project administration, funding acquisition, and intellectual input. All authors contributed to the article and approved the submitted version.
