# Supplementary material for: 1,4-dihydroxy quininib modulates the secretome of uveal melanoma tumour explants and a marker of oxidative phosphorylation in a metastatic xenograft model
Source: Front Med (Lausanne). 2023 Jan 9;9:1036322. doi: 10.3389/fmed.2022.1036322 (PMC9868667; doi:10.3389/fmed.2022.1036322)

1,4-dihydroxy quininib modulates the secretome of uveal melanoma tumour explants and a marker of oxidative phosphorylation in a metastatic xenograft model

Kayleigh Slater[1,2], Rosa Bosch[3], Kaelin Francis Smith[1,2], Chowdhury Arif Jahangir[1,2], Sandra Garcia-Mulero[4,5], Arman Rahman[1,2], Fiona O'Connell[6], Josep M. Piulats[7], Valerie O' Neill[8], Noel Horgan[8], Sarah E. Coupland[9,] Jacintha O'Sullivan[6], William M. Gallagher[1,2], Alberto Villanueva[3,10], Breandán N. Kennedy[1,2]*

[1]UCD School of Biomolecular and Biomedical Science, University College Dublin, Ireland

[2]UCD Conway Institute of Biomolecular and Biomedical Research, University College Dublin, Ireland

[3]Xenopat S.L., Parc Científic de Barcelona, Barcelona, Spain

[4]Unit of Biomarkers and Susceptibility, Oncology Data Analytics Program (ODAP), Catalan Institute of Oncology (ICO), Oncobell Program, Bellvitge Biomedical Research Institute (IDIBELL) and CIBERESP, L'Hospitalet de Llobregat, Barcelona, Spain

[5]Department of Clinical Sciences, Faculty of Medicine and Health Sciences, University of Barcelona, Spain

[6]Department of Surgery, Trinity St. James's Cancer Institute, Trinity Translational Medicine Institute, St. James's Hospital, Dublin 8, Ireland.

[7]Medical Oncology Department, Catalan Institute of Cancer (ICO), IDIBELL-OncoBell, Barcelona, Spain

[8]Royal Victoria Eye and Ear Hospital, Adelaide Road, Dublin, Ireland

[9]Liverpool Ocular Oncology Research Group, Department of Molecular and Clinical Cancer Medicine, Institute of Systems, Molecular and Integrative Biology, University of Liverpool, U.K

[10]Chemoresistance and Predictive Factors Group, Program Against Cancer Therapeutic Resistance (ProCURE), Catalan Institute of Oncology (ICO), Oncobell Program, Bellvitge Biomedical Research Institute (IDIBELL), L'Hospitalet del Llobregat, Barcelona, Spain.

SUPPLEMENTARY MATERIAL

Supplementary Figure 1. Control tissue and clinical characteristics of patients with primary UM.

(**A**) Colorectal cancer tissue used as positive control to validate $CysLT_1$ antibody staining at a dilution of 1:200. Strong cytoplasmic staining was detected. (**B**) Colorectal cancer tissue used as positive control for $CysLT_2$ antibody staining as a dilution of 1:500. Intermediate cytoplasmic staining was detected. (**C**) Colorectal cancer tissue negative control (omission of primary antibody). **(D**) Clinical characteristics of the 68 patients from Spain presenting with UM analysed for $CysLT_1$ expression. **(E)** Clinical characteristics of the 48 patients from Spain presenting with UM analysed for $CysLT_2$ expression.

Supplementary Figure 2. Inflammatory and angiogenic factors unchanged after drug treatment in tumour conditioned media of *ex vivo* primary UM explants.

Treatment with all drugs tested, quininib, 1,4-dihydroxy quininib or dacarbazine, did not significantly alter the secretion of (**A**) IFN-γ, (**B**) IL-10, (**C**) IL-12p70 (**D**) IL-4, (**E**) IL-6, (**F**) IL-8, or (**G**) IL-1β (**H**) bFGF, (**I**) Flt-1, (**J**) PlGF (**K**) Tie-2, (**L**) VEGF-A, (**M**) VEGF-C, or (**N**) VEGF-D in *ex vivo* primary UM tumour explants following 72-hour treatment. Conditioned media were collected and analysed by ELISA (n = 11). All secretions were normalised to total protein content and results calculated as fold change compared to vehicle control. Statistical analysis was performed by ANOVA with Dunnett's post hoc multiple comparison test. Error bars are the mean ± S.D.

Supplementary Table 1.

Clinical characteristics of the primary UM patients who donated tumour samples post eye enucleation.

Supplementary Figure 3. Treatment with $CysLT_1$ antagonists does not significantly alter the expression of COX-2, Bcl-2, or Calpain-2 in Mel285 and OMM2.5 cells.

(**A-F**) Representative western blots and densitometry analysis of UM cell lines treated with CysLT receptor antagonists or dacarbazine. Treatment with CysLT receptor antagonists or dacarbazine for 24 hours does not significantly alter COX-2 expression normalized to β-actin in Mel285 (**A**) or OMM2.5 (**B**) cells. Treatment with CysLT receptor antagonists or dacarbazine for 24 hours does not significantly alter Bcl-2 expression normalized to β-actin in Mel285 (**C**) or OMM2.5 (**D**) cells. Treatment with CysLT receptor antagonists or dacarbazine for 24 hours does not significantly alter Calpain-2 expression normalized to α-tubulin in Mel285 (**E**) or OMM2.5 (**F**) cells. Immunoblotting was conducted three separate times using protein lysates from three individual experiments. Statistical analysis was performed by ANOVA with Dunnett's post hoc multiple comparison test. Error bars are mean ± S.E.

Supplementary Figure 4. Tumours with UM characteristics grew in the liver of all study mice.

Representative tumours and corresponding H&E sections shown for mice treated with vehicle (**A**), 1,4-dihydroxy quininib (Q7) (**B**), or dacarbazine (**C**). In general, a single large tumour mass was observed. However, in some cases, many small cancer nodules were observed throughout the liver. Red arrows indicate the primary liver tumour. White arrows indicate small cancer nodules throughout the liver. H&E analysis of liver tumours confirmed the presence of a UM cell phenotype. (**B**) Brown/black deposits of 1,4-dihydroxy quininib can be observed.

Supplementary Figure 5. Metastases to the peritoneal wall or mesentery were significantly lower in 1,4-dihydroxy quininib and dacarbazine treatment groups.

All treated xenograft animals were assessed for metastases within the peritoneal cavity. (**A**) The number of animals with metastases to the peritoneal wall was significantly lower ($p < 0.0001$) in the 1,4-dihydroxy quininib and dacarbazine treated groups versus vehicle. (**B**) The number of animals with metastases to the mesentery was significantly lower ($p < 0.0001$) in the dacarbazine

treated group compared to both the vehicle and 1,4-dihydroxy quininib treated groups. (**C**) There were no significant differences across treatment groups in the number of animals with metastases to the diaphragm. Representative images show metastatic tumours present in the peritoneal wall (**D**), mesentery (**E**), and diaphragm (**F**). Tumours are indicated with a white arrow. Given that the peritoneal wall, mesentery, and diaphragm can all be considered peritoneal metastasis, we analysed the data based on whether metastases were present at all sites (**G**) and at 1 or greater than 1 site (**H**). (**G**) The number of animals with metastases at all sites was significantly lower in the dacarbazine treated group compared to both the vehicle and 1,4-dihysroxy quininib treated groups ($p < 0.0001$). (**H**) The number of animals with metastases at greater than 1 site was significantly higher in the vehicle group compared to both the 1,4-dihydroxy quininib and dacarbazine treated groups ($p < 0.0001$). Statistical analysis was performed using a Fisher's exact to assess differences between two groups. *** $p < 0.001$, **** $p < 0.0001$.

Supplementary Figure 6. UM patients from The Cancer Genome Atlas (TCGA) with high *BAP1* and low *ATP5F1B* expression have a significantly better disease-free survival.

(**A**) Patients from the TCGA-UM dataset were categorised into those that received a diagnosis of primary UM, but their disease had not progressed (non-relapsed) versus those who had developed recurrent disease (relapsed) and stratified by *BAP1* and *ATP5F1B* expression. There is a significant difference in disease-free survival between patients with high *BAP1* expression and low *ATP5F1B* expression versus those with high *BAP1* expression and high *ATP5F1B* expression ($p = 0.02$), those with low *BAP1* and high *ATP5F1B* expression ($p = 0.002$), and those with low *BAP1* and low *ATP5F1B* expression ($p = 0.03$). There is also a significant difference in disease-free survival between those with low *BAP1* and low *ATP5F1B* versus those with low *BAP1* and high *ATP5F1B* expression ($p < 0.001$). (**B)** There is a significant difference in overall survival between patients with high *BAP1* and low *ATP5F1B* expression and those with low *BAP1* and low *ATP5F1B* expression ($p = 0.008$), and those with low *BAP1* and high *ATP5F1B* expression ($p = 0.006$). There is no significant difference in overall survival between patients with high *BAP1* and low *ATP5F1B* expression and those with high *BAP1* and high *ATP5F1B* expression ($p = 0.07$). Both Log-rank p-values and Cox p-values were calculated and are displayed.

Supplementary Figure 1.

A

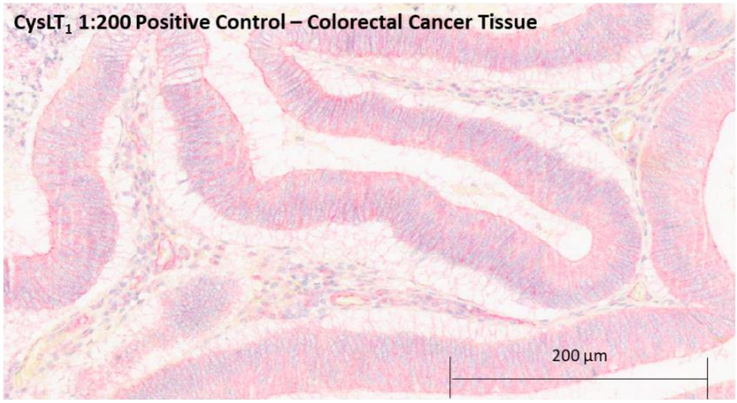

B

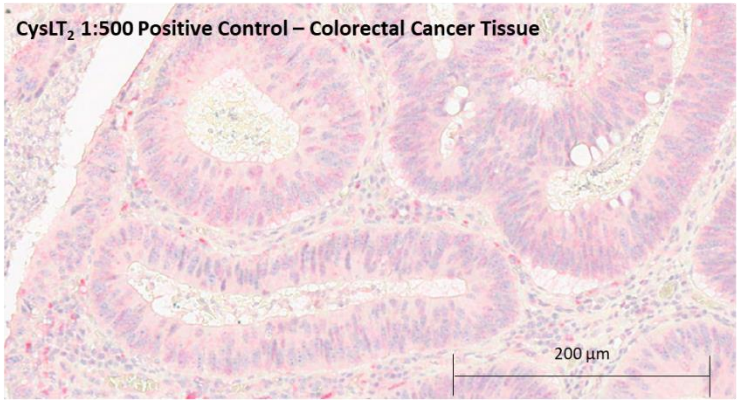

C

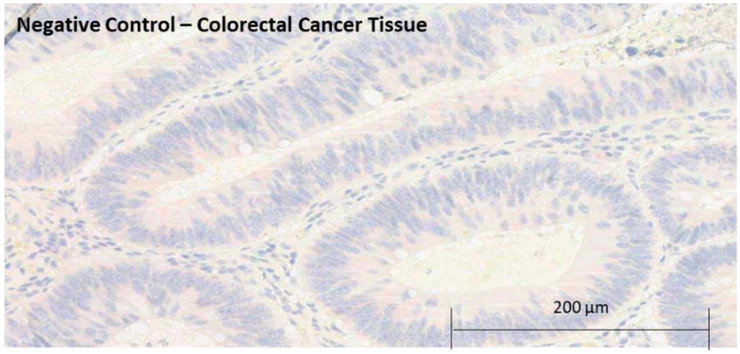

D

CysLT$_1$	Age at primary management	Survival (Years) or to study end
Mean	62	6.22
Median	61	5
Range	64	27.5
Maximum	96	28
Minimum	32	0.5

E

CysLT$_2$	Age at primary management	Survival (Years) or to study end
Mean	62.9	6.1
Median	62	5
Range	55	27.2
Maximum	91	28
Minimum	36	0.8

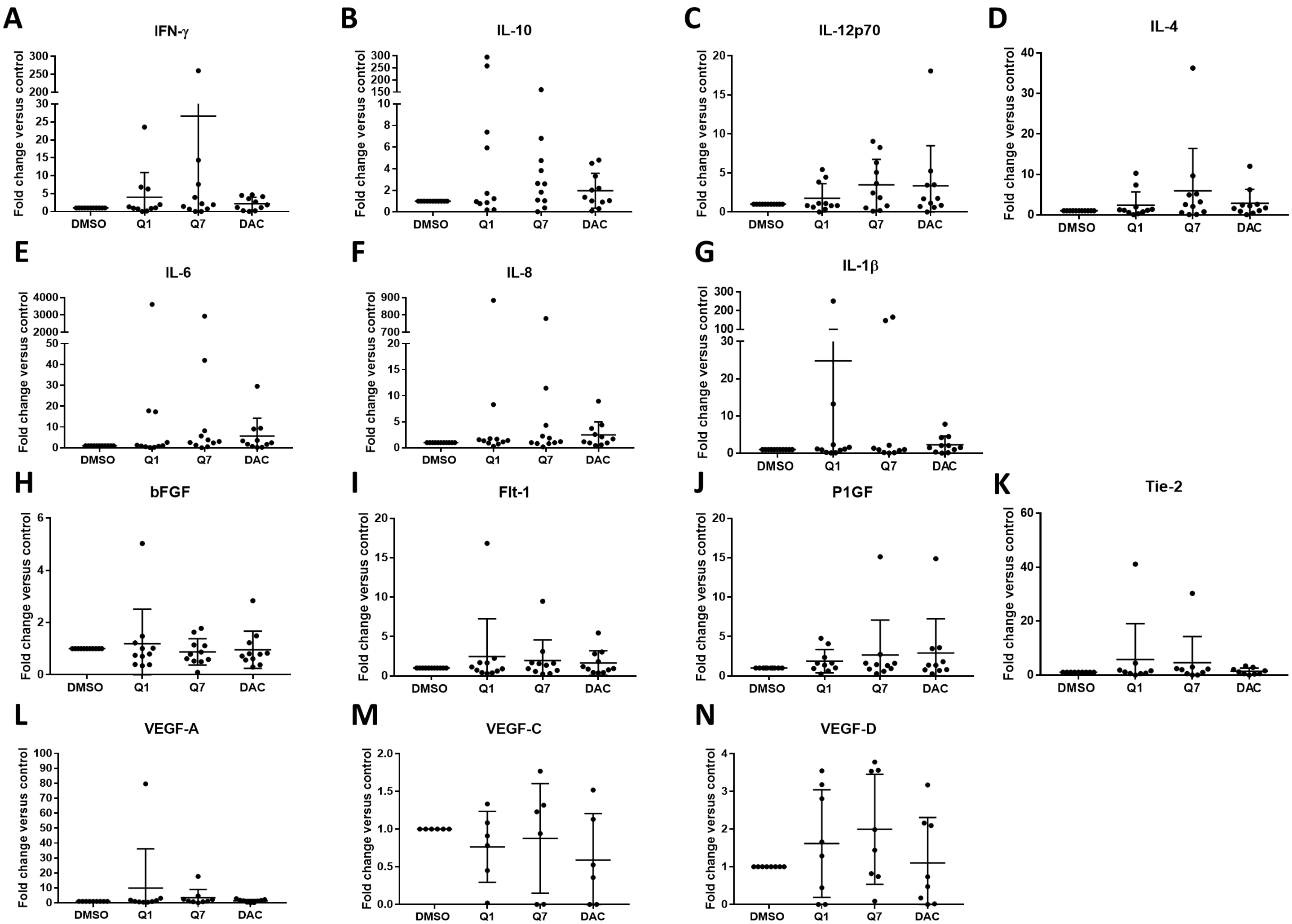
A
IFN-γ
B
IL-10
C
IL-12p70
D
IL-4
E
IL-6
F
IL-8
G
IL-1β
H
bFGF
I
Flt-1
J
P1GF
K
Tie-2
L
VEGF-A
M
VEGF-C
N
VEGF-D
Fold change versus control
DMSO
Q1
Q7
DAC

Supplementary Table 1.

	Age	Largest ultrasound diameter	Largest ultrasound height	Tumour thickness
Mean	56	12.6	10.8	6.9
Median	56	11.3	9.4	6.5
Range	49	10.2	7.6	7.5
Maximum	72	18	15.6	11.3
Minimum	33	7.8	8	3.8

Clinical feature	Present	Absent
Extraocular extension	1	10
Ciliary body involvement	1	10
Epithelioid cell involvement	4	7
Monosomy 3	3	8
Chromosome 8 alteration	6	5

Supplementary Figure 3.

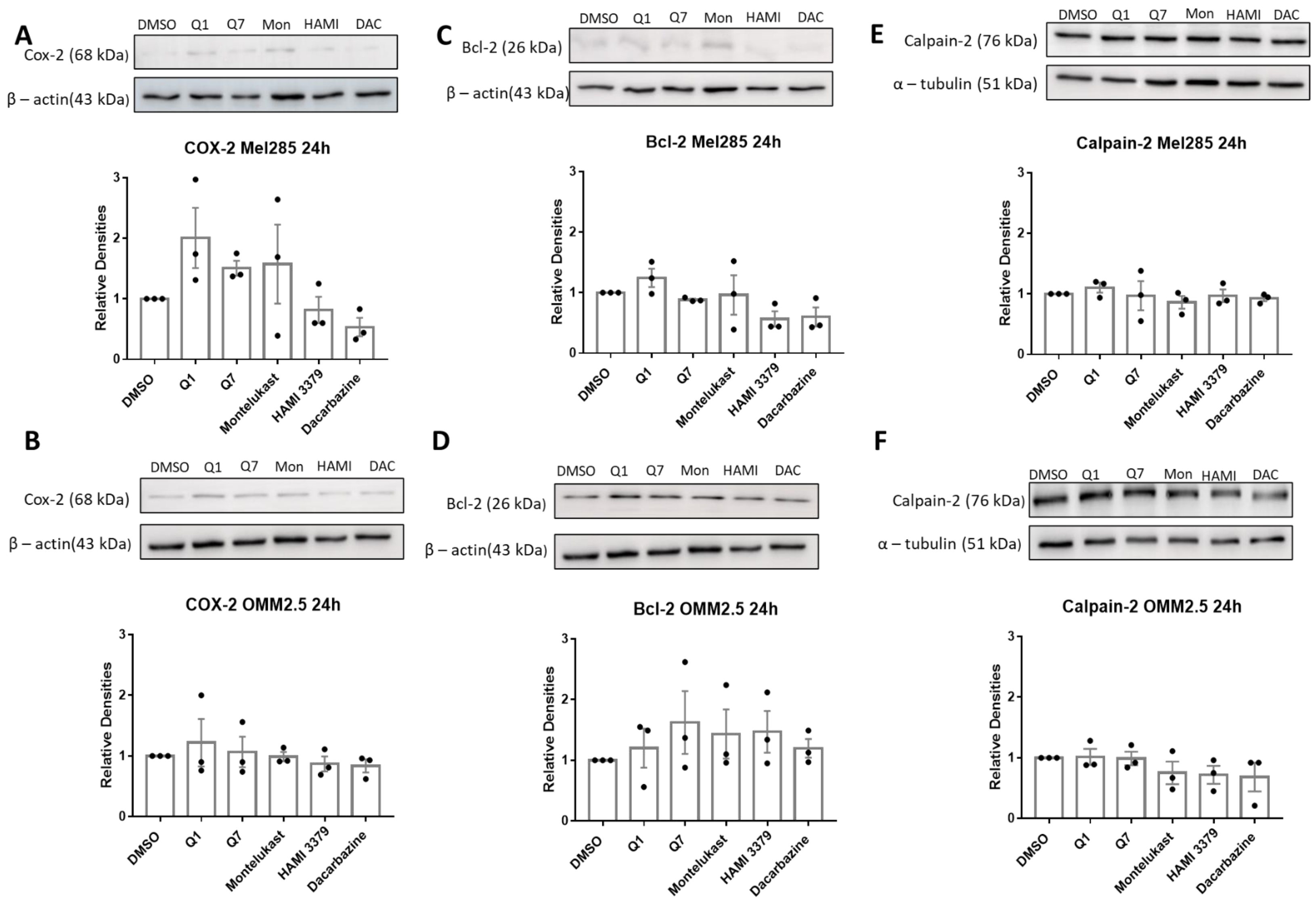

Supplementary Fig 4.

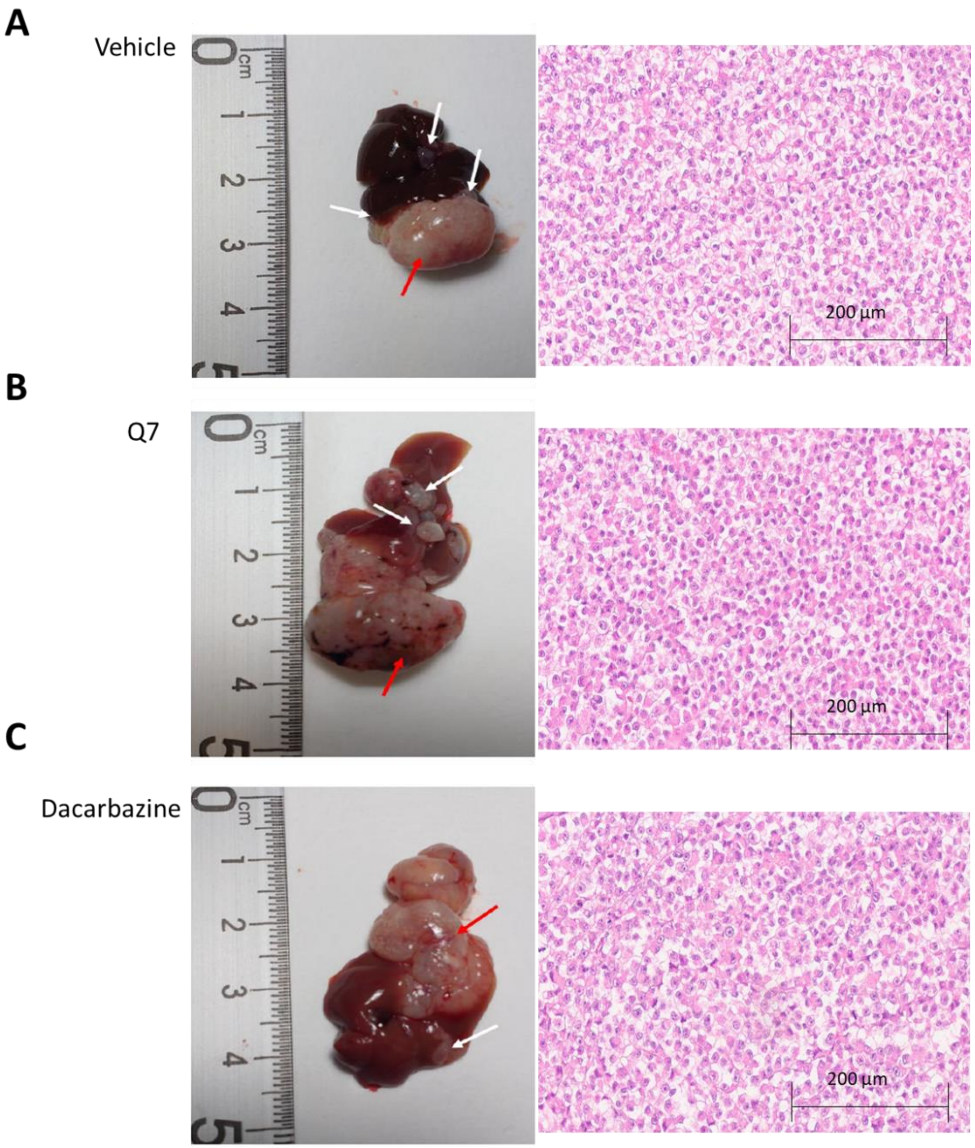

Supplementary Fig 5.

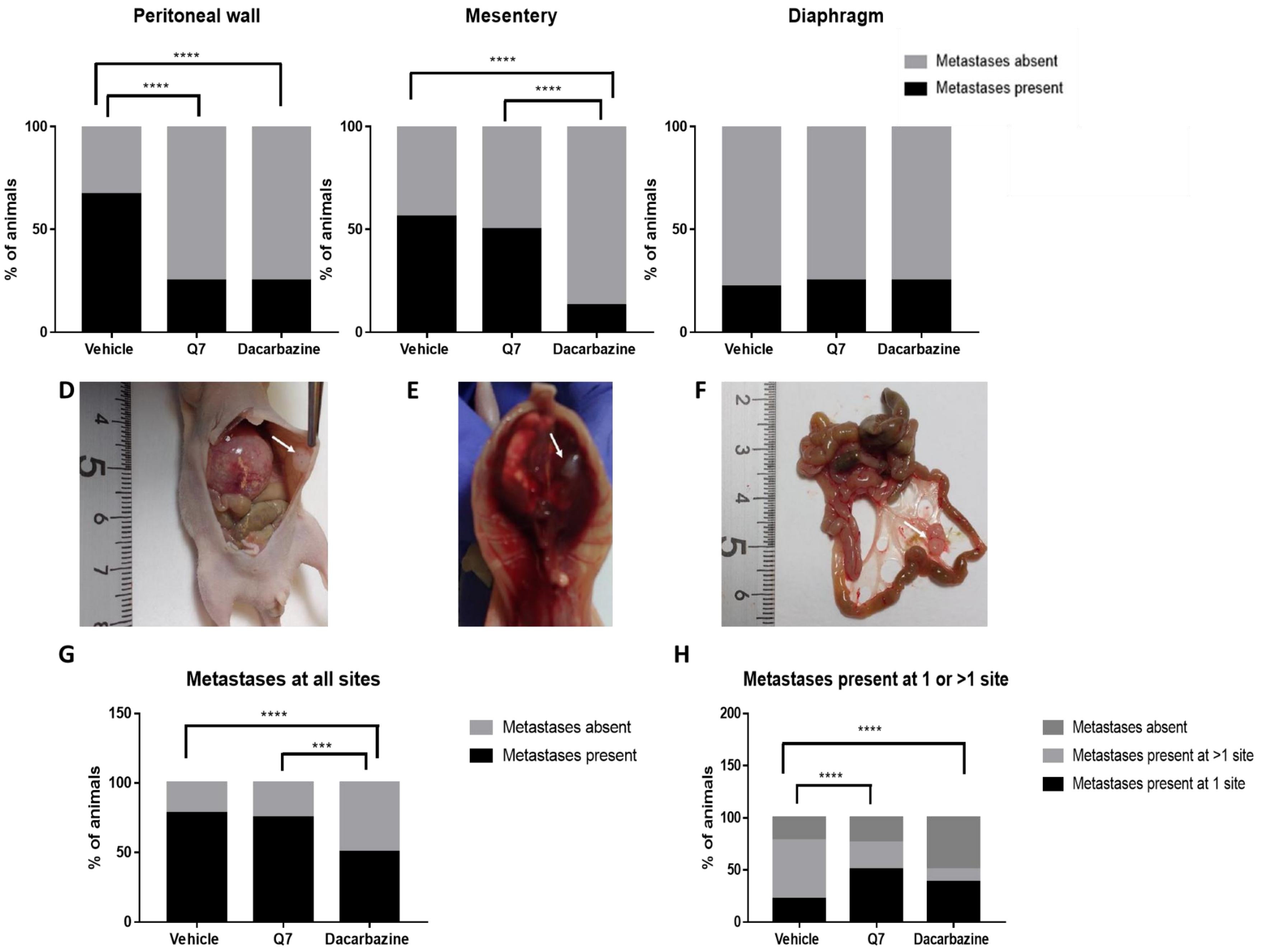

Supplementary Figure 6.

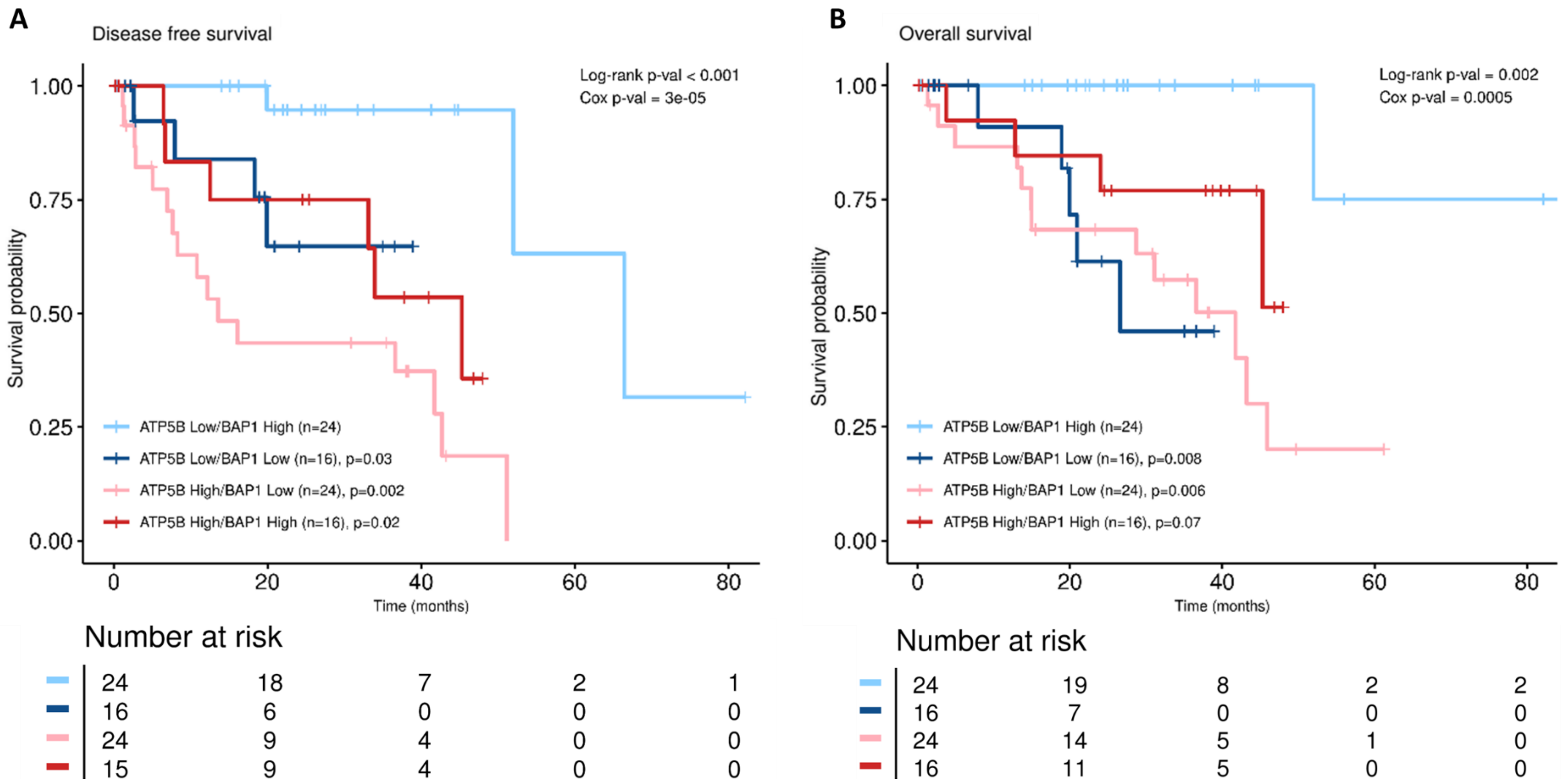

Supplement: Supplementary file 1 [file Presentation_1.pdf]
